# CNN stability training improves robustness to scanner and IHC-based image variability for epithelium segmentation in cervical histology

**DOI:** 10.3389/fmed.2023.1173616

**Published:** 2023-07-05

**Authors:** Felipe Miranda Ruiz, Bernd Lahrmann, Liam Bartels, Alexandra Krauthoff, Andreas Keil, Steffen Härtel, Amy S. Tao, Philipp Ströbel, Megan A. Clarke, Nicolas Wentzensen, Niels Grabe

**Affiliations:** ^1^Institute of Pathology, University Medical Center Göttingen UMG, Göttingen, Germany; ^2^Hamamatsu Tissue Imaging and Analysis Center (TIGA), BIOQUANT Center, Heidelberg University, Heidelberg, Germany; ^3^Medical Oncology Department, National Center for Tumor Diseases (NCT), Heidelberg, Germany; ^4^Medical Faculty, Center of Medical Informatics and Telemedicine (CIMT), University of Chile, Santiago, Chile; ^5^Division of Cancer Epidemiology and Genetics, US National Cancer Institute (NCI), Bethesda, MD, United States

**Keywords:** digital pathology, deep learning, robustness, image variability, stability training, cervical intraepithelial neoplasia

## Abstract

**Background:**

In digital pathology, image properties such as color, brightness, contrast and blurriness may vary based on the scanner and sample preparation. Convolutional Neural Networks (CNNs) are sensitive to these variations and may underperform on images from a different domain than the one used for training. Robustness to these image property variations is required to enable the use of deep learning in clinical practice and large scale clinical research.

**Aims:**

CNN Stability Training (CST) is proposed and evaluated as a method to increase CNN robustness to scanner and Immunohistochemistry (IHC)-based image variability.

**Methods:**

CST was applied to segment epithelium in immunohistological cervical Whole Slide Images (WSIs). CST randomly distorts input tiles and factors the difference between the CNN prediction for the original and distorted inputs within the loss function. CNNs were trained using 114 p16-stained WSIs from the same scanner, and evaluated on 6 WSI test sets, each with 23 to 24 WSIs of the same tissue but different scanner/IHC combinations. Relative robustness (rAUC) was measured as the difference between the AUC on the training domain test set (i.e., baseline test set) and the remaining test sets.

**Results:**

Across all test sets, The AUC of CST models outperformed “No CST” models (AUC: 0.940–0.989 vs. 0.905–0.986, *p* < 1e − 8), and obtained an improved robustness (rAUC: [−0.038, −0.003] vs. [−0.081, −0.002]). At a WSI level, CST models showed an increase in performance in 124 of the 142 WSIs. CST models also outperformed models trained with random on-the-fly data augmentation (DA) in all test sets ([0.002, 0.021], *p* < 1e-6).

**Conclusion:**

CST offers a path to improve CNN performance without the need for more data and allows customizing distortions to specific use cases. A python implementation of CST is publicly available at https://github.com/TIGACenter/CST_v1.

## Introduction

Advances in both digital pathology and the application of Deep Learning (DL) on Whole Slide Images (WSI) are rapidly progressing, and accordingly, multiple applications are being developed (1). Convolutional Neural Networks (CNNs) have shown to be highly effective in performing tasks such as nuclei classification and segmentation (2, 3), tissue type segmentation (4), and WSI-level or lesion-level diagnosis (5, 6), even exceeding human performance for some diagnostic tasks in controlled scenarios, such as the detection of lymph node metastases in breast cancer (7–9).

One of the main limitations of DL, and an ongoing challenge for its application in healthcare, is the poor performance of CNNs trained on single-source data when used on new data sets (10). Slight variations in color, noise, contrast, compression loss, rotation and other properties can cause DL models to misclassify (11, 12), thus showing a lack of *robustness to image variability*. In digital pathology, this variability can be caused by the use of different scanners, sample preparation and even environmental factors. Evidence shows that DL models underperform on WSIs from different scanners compared to those used to generate the training sets (13, 14). DL models evaluating Immunohistochemistry (IHC) stains and rare-event detection might especially suffer from slide heterogeneity (15). Since IHC biomarkers are evaluated for clinical decision making, the robustness of the classification is of critical importance for a clinical use of DL models.

We here propose an adaptation of CNN Stability Training (CST) (16) for WSIs for improving robustness to biological and technical variations (samples, sample processing and optical image acquisition) and for evaluating IHC staining patterns. CST was originally designed to increase DL model robustness to image distortions like cropping, JPEG compression and image resizing. Here we extend this initial idea further and apply it to two major challenges of Whole Slide Imaging which are (1) the use of different scanners and (2) the use of different IHC stains. With CST, DL models are trained to maximize agreement between the outputs for pairs of original and artificially distorted images through a parallel augmentation. Distorted images are randomly generated on the fly during training, through composition of artificial distortions to emulate naturally occurring medical image variations: blurriness, color, brightness and contrast. Implementing the CST approach in software code requires encapsulating any given CNN network structure into a two-input-channel frame layout. This frame layout is highly customizable, and allows to adapt any CNN architecture to specific challenges of robustness. CST shares conceptual similarities with supervised contrastive learning (17, 18), since both attempt to increase agreement between outputs on original and artificially distorted images. While recent contrastive learning work usually targets agreement between feature representations, CST directly targets agreement between CNN likelihoods.

We have applied CST for the automatic segmentation of epithelium in IHC-stained cervical WSIs. IHC-staining creates a patterned appearance of the biomarkers under study over a homogenous hematoxylin background. The pattern is highly relevant for the diagnostic evaluation. In combination with technical distortions arising from variations in the sample itself, the sample preparation and/or its scanning can make a robust segmentation very challenging. Automatic segmentation is a prerequisite for the automatic quantification of IHC expression in subareas of WSIs, supporting a more objective computationally-based diagnosis of cervical precancer (Cervical Intraepithelial Neoplasia grade 2 and 3, CIN2 and CIN3) and early detection of cervical cancer. We evaluated and compared DL models trained with and without CST, on WSIs from three different scanner models, two of which were not used for training. This allowed us to evaluate robustness to distortions caused by the use of different scanners and, therefore, the potential benefit of using CST.

Human papillomavirus (HPV) testing has been approved for primary cervical cancer screening as a rapid genetic testing for Cervical Intraepithelial Neoplasia (CIN) in its different grades. HPV is the main medical cause for CIN and cervical cancer and is transmitted by skin-to-skin or mucosa-to-mucosa contact. The virus reaches and infects germinal cells in the basal layer of the epithelium, usually near the transformation zone. Some HPV infections are more persistent, making them harder to eradicate by the body’s immune system. The extended duration of these infections causes the proliferation of abnormal cells. As the proliferation of abnormal cells persists, almost the whole thickness of the epithelium becomes neoplastic. p16^INK4a^ (short: p16) has an important role in cell cycle regulation (19) as it is upregulated Human Papilloma Virus (HPV) infection resulting in an over activation of the cell proliferation (20). HPV further additionally interferes with the cell death functions causing affected epithelial cells to become neoplastic, and leading eventually to the development of CIN. The cell cycle regulator p16 serves as an indirect marker for this HPV oncogene activity (20). p16 is used in IHC assays like the CINtec Kit (Roche) for the diagnosis of CIN and has been shown to reduce an otherwise high inter-observer variability of cervical histology (19–25). The tissue frequently reacts to this transformation with an immune cell infiltration which varies for individual patients. The strength of this immune response is thus considered potentially indicative of patient prognosis. CD3 and CD8 T-cell co-receptors are histological hallmarks of such immune cell infiltration which allow to potentially predict prognosis or eventually response to cancer treatment (26–28). The analysis of different IHC such as p16, CD3, and CD8 can contribute to a more precise diagnosis and prognosis of the disease. Therefore, having CNNs that are robust to different scanners and to different IHC stains is a requirement for the large-scale processing of WSIs.

The biomarker p16 may stain larger areas of the epithelium with an eventual gradient of intensity toward the tissue surface. The staining pattern is indicative of the actual diagnosis and thus a strong heterogeneity of the spatial expression pattern of p16 is core to its nature. In contrast, CD3 and CD8 show a cellular and membrane-type like pattern. With the purpose of evaluating robustness to variations in IHC patterns, we trained CNNs with p16-stained WSIs only, and evaluated on WSIs stained with p16, CD3 and CD8.

The main contributions of this work are the following:

- We propose a novel stability framework setup to train CNNs which allows an increase in robustness to image distortions using CST, in the context of digital pathology.
- We show that the segmentation accuracy of a DL model which was trained on examples of one scanner model may significantly drop when applied on a different scanner model of the same manufacturer (here Hamamatsu Photonics Nanozoomer).
- We propose a set of domain-specific distortions for image properties (i.e., contrast, color, brightness, blurriness) for the implementation of CST.
- Using the p16, CD3 and CD8 biomarkers, we validate how far CST can improve segmentation performance on WSIs from different scanner models, and may further improve performance on WSIs from the same scanner.
- We benchmark CST with traditional on-the-fly data augmentation using the same domain-specific distortions for image properties.

## Materials and methods

### Datasets

We used 256 WSIs from tissue samples from the Study to Understand Cervical Cancer Early Endpoints and Determinants (SUCCEED) conducted by the Division of Cancer Epidemiology and Genetics of the National Cancer Institute (DCEG-NCI) in collaboration with the University of Oklahoma (21, 22). SUCCEED was a cross-sectional study of women 18 years of age or older with an abnormal Pap smear who were referred to colposcopy or treatment at the University of Oklahoma (OUHSC) between 2003 and 2011. Written informed consent was obtained from all women enrolled in the study, and Institutional Review Board approval was provided by OUHSC and the US National Cancer Institute (NCI). The biopsy specimens were collected at the colposcopy visit among women with abnormal screening results. Sections from paraffin-embedded tissue blocks were cut at the University of Oklahoma. Sections were fixed onto glass slides and stained either for p16 (monoclonal antibody clone E6H4, no dilution, Roche), CD3 (monoclonal antibody clone SP7, dilution 1:100, Diagnostic BioSystems) or CD8 (monoclonal antibody clone SP16, dilution 1:100, Diagnostic BioSystems) at the University of Heidelberg. These slides were then digitized into WSIs using three different whole-slide scanner models from Hamamatsu: NanoZoomer-HT (HT), NanoZoomer-XR (XR) and NanoZoomer S360 (S360). In order to induce blur, an additional set of WSIs was generated intentionally out-of-focus, using offsets of 5–20 μm of the microscopic objective. Finally, epithelium regions within the WSIs were manually annotated by researchers at the DCEG-NCI using the software NDP.view2 from Hamamatsu. All slides were scanned at 20× magnification (1 pixel = 0.45 × 0.45 μm^2^).

**Figure 1:**
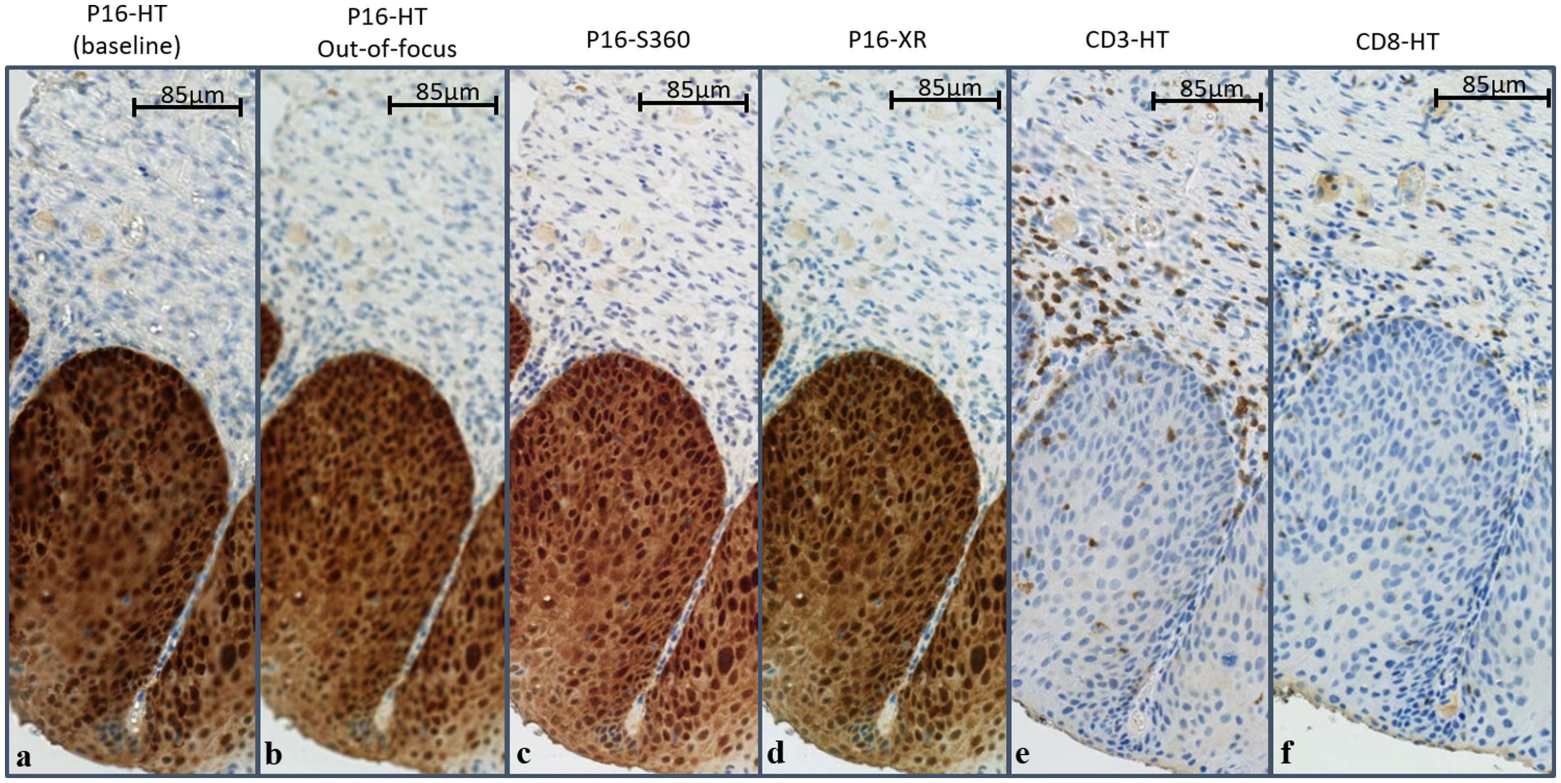
Example WSIs showing image variability. Slides were stained for p16 **(A–D)**, CD3 **(E)** or CD8 **(F)**, and scanned with the NanoZoomer HT **(A,B,E,F)**, XR **(D)** or S360 **(C)**. Scanner-caused image variability (between **A–D**) can be observed as variations in color, blur or contrast, while staining-caused image variability (between **A,E,F**) can be observed as variations in color patterns, associated with the biomarker expression. p16 expression can be observed on neoplastic cells within the epithelium, while CD3 and CD8 expression can be observed as scattered brown stains over CD3+ and CD8+ T cells, mostly in the stroma surrounding the neoplastic epithelium. Slight morphological differences can be observed between WSIs stained for CD3, CD8 and p16, as these constitute different slices of the same tissue. WSIs were scanned at 20× resolution (1 pixel = 0.45 × 0.4 μm^2^).

The WSIs were separated into one training set and six test sets, as detailed in Table 1. The dataset generation and curation process is presented with more detail in Supplementary material. The training set corresponds to 114 WSIs of p16-stained tissue slides from different patients, scanned with the HT. The six test sets correspond to WSIs of slides with varying IHC stains and/or digitized with different scanner models. Test sets with p16 staining were generated by scanning the same glass slides using different scanners (resulting in the datasets *P16-HT-baseline, P16-XR* and *P16-S360*), and scanning intentionally out-of-focus (resulting in the dataset *P16-HT-oof*). The test sets with CD3 and CD8 staining were generated using different sections of the same tissue block used for p16 staining, which is why these WSIs look morphologically similar but not identical between each other (see Figure 1). All WSIs from the test sets come from different tissue samples than those used for the training set. The manual annotations were used as labels to train the CNNs with the training set, and as the gold standard to evaluate CNN performance with the test sets.

**Table 1:** Datasets used for training and testing.

Purpose	Stain-scanner abbrev.	Type of staining	Scanner	No of WSIs	No of epithelium tiles	No of non-epithelium tissue tiles
Training	P16-HT train	P16	NanoZoomer-HT	114	405.992^a^	607.627
Testing	P16-HT baseline	P16	NanoZoomer-HT	24	12.601^b^	66.221^b^
	P16-HT-oof	P16	NanoZoomer-HT (WSIs scanned intentionally out-of-focus)	23	9.792^b^	54.164b
	P16-XR	P16	NanoZoomer-XR	24	11.416^b^	58.259^b^
	P16-S360	P16	NanoZoomer S360	23	11.132^b^	59.239^b^
	CD3-HT	CD3	NanoZoomer-HT	24	11.297^b^	69.095^b^
	CD8-HT	CD8	NanoZoomer-HT	24	11.073^b^	71.365^b^

All DL models were trained with p16 slides scanned with the NanoZoomer-HT. These were tested on datasets generated using different scanners (*P16-HT baseline, P16-XR and P16-S360*), intentionally out of focus (*P16-HT-oof*) and using different IHC stains (*CD3-HT* and *CD8-HT*). ^a^8-fold data augmentation was applied by means of 90° rotations and flipping to correct class imbalance. ^b^The reason for the difference in the number of tiles between test sets is the preprocessing method, where background or excessively blurred tiles were discarded from the set. Due to variations in focus and/or contrast, some tiles may have been discarded as background in some test sets but not in others. This is especially evident for the *P16-HT-oof* test set.

Figure 1 illustrates the naturally occurring variability caused by the use of different scanner models, the lack of focus and the use of different IHCs. Distortions caused by different scanners or machine operators can be seen as changes in color, contrast, brightness of blur. On the other hand, distortions caused by the IHC stain can be defined as variations in color patterns, as these biomarkers may express in different colors and over different tissue regions. The p16 IHC is expressed as a brown stain covering a large region of the epithelium, while the CD3 and CD8 IHC are expressed as brown blobs scattered around and inside the epithelium.

Before being used for training and testing, all WSIs were preprocessed into 128x128px tiles without overlap. Tiles corresponding to background (i.e., WSI area without tissue) were removed from all datasets in a two-step process. First, a histogram threshold was applied on a grayscale version of each tile, by setting a maximum number of pixels within a specific range from the intensity median. Tiles not passing this criterion were labeled as background and removed. Second, a laplacian filter was applied to the remaining tiles for edge detection. Tiles with a lower edge sharpness than the set criterion were also labeled as background and removed. Supplementary Figures 1–8 describing the process are available in Supplementary material. Next, tiles were labeled 1 for “epithelium” tiles (i.e., within the manually annotated region of the WSI) or 0 for “non-epithelium” (i.e., outside of the manually annotated region of the WSI). Finally, an 8-fold data augmentation was applied only on the “epithelium” tiles of the training set to reduce the class imbalance by applying three rotations (90°, 180°, and 270°) and flipping, resulting in over 1 million tiles for training. The 8-fold value was chosen over a lower fold due to the large gap in the ratio between “non-epithelium” and “epithelium” tiles, of approximately 11:1.

### CNN stability training for robustness

The goal of CNN stability training (CST) is to reduce the variability of the softmax outputs caused by input image distortions while training a CNN. In other words, if an image *x* and its distorted version *x* ${}^{\prime }$ are similar, then the output $f_{\theta }\left(x\right)$ should be similar to $f_{\theta }\left(x^{\prime }\right)$ as well, given the DL model’s set of trainable parameters *θ*. For this purpose, the CST approach incorporates a “stability component” in the calculation of the objective loss function, which aims to maximize agreement between the softmax of an image and its distorted version. Specifically, given an image *x* and its distorted version *x* ${}^{\prime }$ , the loss function is calculated as a composition of two distance measures:


(1) $$L(x,x^{\prime },\hat{y};\theta )=L_{0}(x,\hat{y};\theta )+\alpha \cdot L_{stab}(x,x^{\prime },\theta )$$

Where $\hat{y}$ represents the one-hot encoded ground truth for image *x*. $L_{0}$ is the distance between the prediction of the original image and its true label, which forces the DL model to correctly predict image classes during training. The stability component $L_{stab}$ is the distance between the prediction of the original image and the prediction of the distorted image. This component constrains the objective loss function and forces the DL model to minimize the distance between the prediction for *x* and *x’* during training. The parameter *α* determines the weight of the stability component in the objective loss function. The value of *α* has to be fine-tuned, and will differ depending on the dataset and the level of distortion applied to it. Selecting α=0 is equivalent to training without CST.

**Figure 2:**
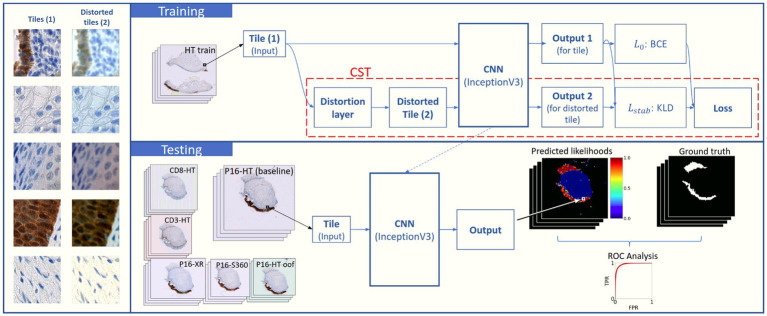
Schema for the training process with CST and the testing setup. Multiple models were trained using different Distortion Combinations (DC) and α values (where α = 0 means no CST; training). During the training process, input tiles pass through a distortion layer which generates a distorted version of the tiles. Both the original and the distorted tiles are fed to an InceptionV3 CNN in parallel, which outputs a likelihood for each, Output 1 and Output 2. Loss components $L_{0}$ and $L_{stab}$ are calculated using BCE (Eq. 2) and KLD (Eq. 3), respectively. An ensemble loss function (Eq. 1) is calculated with both values. The trained DL models are evaluated on the baseline testing set (P16-HT-Baseline) and on the remaining five testing sets with real-world distortions (testing). Tile-wise and WSI-wise analyses are performed over the model outputs, and performance is compared through the analysis of the ROC curves and the calculation of AUC and rAUC.

For mini-batch training with *N* images per batch, the distance $L_{0}$ is calculated as the Binary Cross-entropy between the DL model’s output for the batch *X* and its respective set of true labels, $\hat{Y}$ :


(2) $$L_{0}(X,\hat{Y};\theta )=-\frac{1}{N}\sum_{i=1}^{N}\sum_{c=1}^{C}{\hat{y}}_{i,c}\cdot \left(P\left(y_{i,c}|x_{i};\theta \right)\right)\;$$

where ${\hat{y}}_{i,c}\in \hat{\;Y}$ is the ground truth regarding class *c* for image $x_{i}\in X$ (i.e., ${\hat{y}}_{i,c}=1$ if $x_{i}$ belongs to class *c*, 0 otherwise) and $P\left(y_{i,c}\mathrm{|;}x_{i}\mathrm{|;}\theta \right)$ is the likelihood predicted by the DL model for image $x_{i}$ to belong in class *c*. On the other and, the stability component $L_{stab}$ is calculated as the Kullback–Leibler Divergence between batch *X* and the distorted batch *X’* (16, 23):


(3) $$L_{stab}(x,x^{\prime },\theta )=-\frac{1}{N}\sum_{i=1}^{N}\sum_{c=1}^{C}P\left(y_{i,c}|x_{i};\theta \right)\cdot \log\left(\frac{P\left(y_{i,c}|x_{i};\theta \right)}{P\left(y_{i,c}|x\prime_{i};\theta \right)}\right)$$

where $P\left(y_{i,c}\mathrm{|;}x\prime_{i}\mathrm{|;}\theta \right)$ is the likelihood predicted by the DL model for the distorted image $x\prime_{i}$ to belong in class *c*. It can be observed that $L_{stab}$ is not affected by the correctness of the prediction (i.e., how close the likelihood for $x\prime_{i}$ is to class *c*); it is only concerned with how close the likelihoods for $x_{i}$ and $x\prime_{i}$ are to each other. The training process is described in Figure 2.

### Data augmentation

Data augmentation is a method traditionally used in deep learning to increase the size of the training dataset, improve model generalization and avoid overfitting. It consists in applying distortions to the input images, thus artificially attempting to expand the training domain. When applied on-the-fly (instead of being applied *a priori* over the training dataset), random transformations are applied during training, in a similar way as the random transformations applied for CST.

The training setting is the same as a classical setting to train a CNN except for the addition of the same distortion layer used for CST, between the input image and the base architecture. The loss function, $L_{0},$ is calculated as the Binary Cross-entropy between the DL model’s output for the batch X and its respective set of true labels.

### Performance and robustness metrics: AUC and rAUC

The performance metric used for this work was the Area Under the ROC Curve (AUC). We calculated the AUC over the total number of tiles for all test sets to evaluate overall performance. Tiles within the regions manually annotated as “epithelium” were labeled positive, and tiles outside these regions were labeled as negative. These labels were used as the ground truth to build the ROC curves and to calculate AUC.

Regarding robustness, the AUC on the *P16-HT-baseline* test set ( $AUC_{base}$ ) was used as a baseline performance for each DL model, because it has no variability with respect to the training set. To quantify robustness of a DL model on other test sets, we used the difference between $AUC_{base}$ and the AUC of the test sets, $AUC_{testset}$ , which we called $\;rAUC_{testset}$ :


(4) $$rAUC_{testset}=AUC_{testset}-AUC_{base}$$

where $rAUC_{testset}\geq 0$ constitutes perfect relative robustness.

Statistically significant differences between DL model results were calculated over the total number of tiles for each dataset, using the fast version of DeLong’s algorithm for comparing the AUC of correlated ROC curves (24). Additionally, we calculated the AUC at WSI level to analyze performance from a WSI perspective, and created a boxplot to analyze WSI-wise results.

## Experiments and results

We developed 13 models with InceptionV3 as a base architecture. Each model corresponds to an ensemble of its three best performing epochs (i.e., the three epochs with lowest validation BCE during training). The model output is the average of the output of these three epochs.” The combination of multiple model weights trained with different initial weights and data order is called Deep Ensemble, and is used to obtain a better generalization performance and out-of-distribution robustness (19, 20, 25). The results for each independent epoch are provided in Supplementary Table 1.

Each model was trained with a different value for the parameter *α* and a different Distortion Combination (DC), as shown in Table 2. A DC corresponds to a sequence of random artificial distortions applied to an input image to generate a distorted output image. For this experimental setup we proposed and evaluated a set of DCs, as described in the next section, “Distortion Combinations.” One model was trained without CST (model “No CST”) by setting α=0 , which cancels $L_{stab}$ from the loss function, as described in equation (1). Seven models were trained with CST, and an additional five models were trained with on-the-fly data augmentation, using the same distortion combinations from CST. All models were trained on the same training set, *P16-HT Train*.

**Table 2:** List of trained DL models.

Model	α	Distortion combination	Fixed hyper-parameters
No CST	0	–	
CST1	1	1	
CST2	1	2	
CST3	1	3	
CST4	1	4	Epochs: 15
CST5	2	2	Optimizer: SGD
CST6	10	2	Learning rate: 1e − 3
CST7	100	2	Momentum: 0.9
DA1	–	1	Decay: 1e − 6
DA2	–	2	Batch size: 64
DA2_v2	–	2^*^	
DA3	–	3^*^	
DA4	–	4^*^	

All models share a set of fixed hyper-parameters involved in the model optimization process. Each model has a different value for α and/or a different DC. *Within the DCs used to train DA2_v2, DA3 and DA4, the *σ_K_* was set to $\frac{\sigma }{255}$ .

After training the DL models, we evaluated their performance in the task of segmenting epithelium on the six test sets described in the section “Datasets.” Performance was measured as AUC, and robustness was measured using equation (4), which corresponds to the difference between the AUC on each test set and the AUC of the test set *P16-HT-baseline* (i.e., same scanner and IHC as the training set). Figure 2 illustrates the testing process for each DL model.

We used the Keras API implemented on the TensorFlow library. During training, the distortion layer generates distorted tiles from the original input tiles, and both are inputted to the InceptionV3 CNN, which outputs a likelihood for each.

### Distortion combinations

For this work, CST involved the application of distortions in color, contrast, brightness and blur. We selected these image properties based on a visual interpretation of the main differences between WSIs. We proposed four different DCs which apply random distortions of these image properties within certain ranges, as described in Table 3. These ranges increase from DC 1 to DC 4, so the use of a higher DC implies that some images will be highly distorted.

**Table 3:** Distortion combinations that were evaluated.

Image property	Distortion combination (DC)			
	1	2	3	4
Color (red)	*d_R_* ∈ [−5, 5]	*d_R_* ∈ [−5, 5]	*d_R_* ∈ [−20, 20]	*d_R_* ∈ [−20, 20]
Color (green)	0	0	0	*d_G_* ∈ [−20, 20]
Color (blue)	*d_B_* ∈ [−5, 5]	*d_B_* ∈ [−5, 5]	*d_B_* ∈ [−20, 20]	*d_B_* ∈ [−20, 20]
Contrast	*d_C_* ∈ [0.8, 1.2]	*d_C_* ∈ [0.8, 1.2]	*d_C_* ∈ [0.6, 1.6]	*d_C_* ∈ [0.6, 1.6]
Brightness	*d_L_* ∈ [−0.3, 0.3]	*d_L_* ∈ [−0.3, 0.3]	*d_L_* ∈ [−0.5, 0.5]	*d_L_*∈ [−0.5, 0.5]
Blur (Gaussian kernel)	0	kernel size: 5×5 *μ_K_* = 0.0 *σ_K_* ∈ [0.0, 1.0]	kernel size: 5×5 *μ_K_* = 0.0 *σ_K_* ∈ [0.0, 5.0]	kernel size: 9×9 *μ_K_* = 0.0 *σ_K_* ∈ [0.0, 5.0]

The cell color describes the level of distortion: green, yellow, orange, and red cells depict no distortion, low distortion, medium distortion, and high distortion, respectively. Color distortions correspond to an addition of *d_R,_ d_G,_ d_B_* to the respective RGB channels of the image, where *d_R,_ d_G,_ d_B_* are random values between a range determined by the DC. Contrast and brightness distortions correspond to an adjustment of *d* to the contrast and brightness levels of the image.

**Figure 3:**
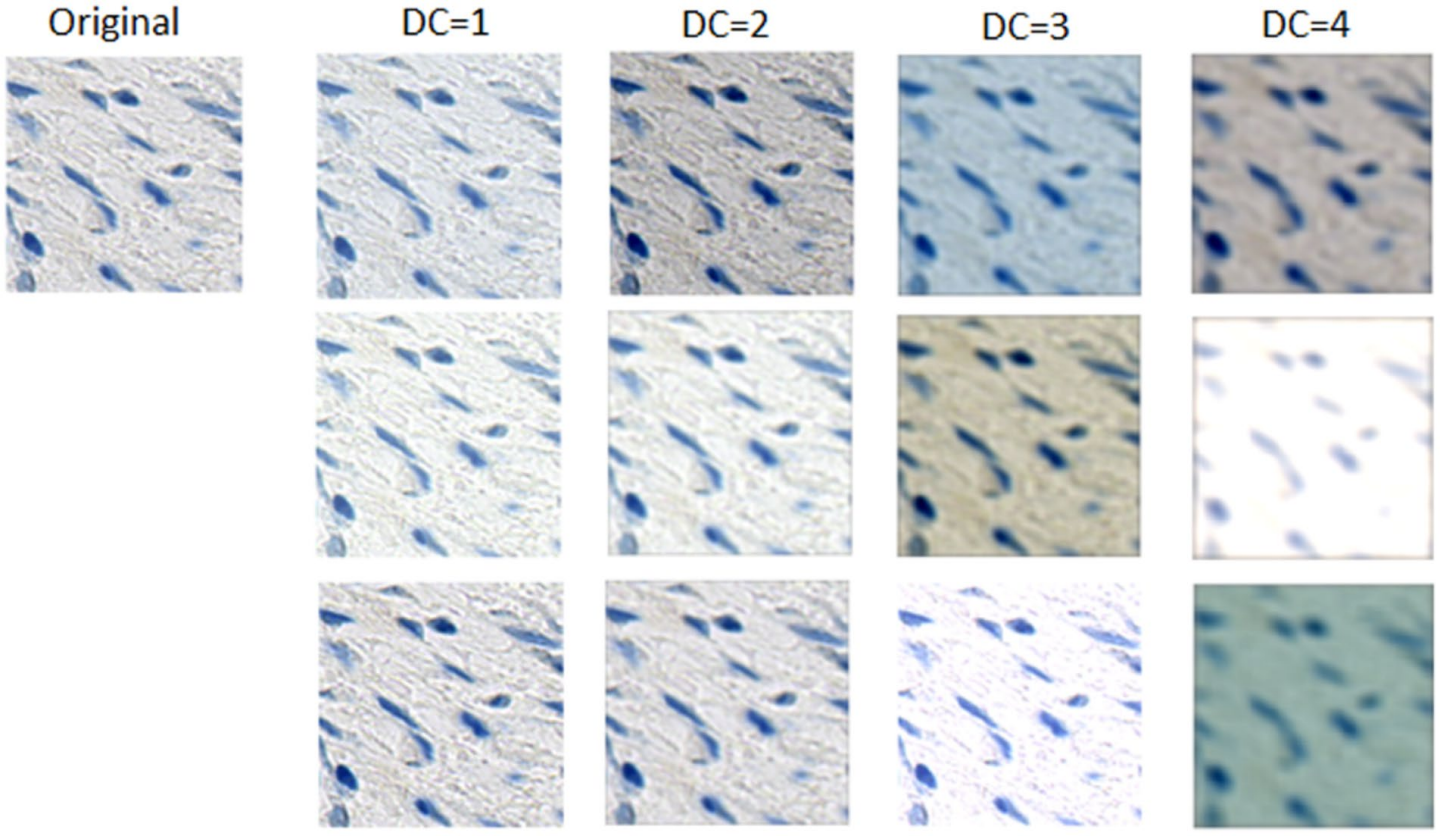
Examples of randomly generated distorted tiles with the four different DCs. Three examples are presented for each DC. Tiles generated with DC = 1 are visually similar to the original, and the difference increases progressively on each combination. Finally, the tiles distorted with DC = 4 seem visually as the most different from the original.

A distortion in color corresponds to the addition of *d_R_*, *d_G_*, and *d_B_* to the RGB values of every pixel in the image. A distortion in brightness corresponds to the addition of $255\cdot d_{L}$ to every pixel RGB value in the image. A distortion in contrast corresponds to the computation of the channel-wise pixel mean $\mu_{C}^{chan}$ and the channel-wise adjustment of each pixel component value *x^chan^* to $\left(x^{chan}-\mu_{C}^{chan}\right)\cdot d_{C}+\mu_{C}^{chan}$ . A distortion in blur corresponds to the convolution of the image with a gaussian kernel centered in 0 (i.e., $\mu_{K}=0$ ), a standard deviation *σ_K_* and a kernel size that depends on the DC. *d_R_*, *d_G_*, *d_B_*, *d_L_*, *d*_*C*,_ and *σ_K_* are random values between ranges determined by the *DC* being used for the training of a specific DL model. During training, every image that is used as input to the network will be distorted with a different value of *d_R_*, *d_G_*, *d_B_*, *d_L_*, *d_C_* and *σ_K_*, so all images will be distorted differently. Since the ranges for all image properties contain a “no distortion” value (i.e., 1 for *d_C_*, 0 for all the others), some images will have a low distortion. Figure 3 illustrates randomly generated examples of distortions obtained using the four different *DCs*. It can be observed that DCs 1 to 3 have no distortion on the green channel. An exploratory analysis of the variability between images showed that the green channel did not significantly change between images, so we decided to omit it with the exception of DC4, which represents the DC with highest distortions.

### Segmentation performance without CST

The purpose of this section was to evaluate the performance of model “No CST” in order to determine a base robustness to real-world distortions without CST. This gave us exemplary insight into the impact that the use of different scanner models and IHC stains has on DL model performance and, therefore, the importance of robustness for applications in digital pathology. Model “No CST” was evaluated on all test sets: *P16-HT-baseline* (i.e., same domain used for training)*, P16-XR, P16-S360, P16-HT-oof, CD3-HT*, and *CD8-HT.*

Segmentation performance for model “No CST” is described in the first row of Table 4. It was calculated as the AUC over the total number of tiles, for each test set. It can be observed that this model performed better on the *P16-HT-baseline* testing set (AUC_base_ = 0.986) than on the remaining five datasets. This result was aligned with our expectations, since this test set belongs to the same domain as the training set (i.e., same scanner, same IHC).

**Table 4:** Performance of the CST models, DA models and “No CST” model on the six test sets.

		P16-HT (baseline)	P16-HT-oof	P16-XR	P16-S360	CD3-HT	CD8-HT	All test sets	
Model	Parameters	AUCbase	AUC_oof_ (rAUCoof)	AUCXR (rAUC_XR_)	AUCS360 (rAUCS360)	AUCCD3 (rAUCCD3)	AUCCD8 (rAUCCD8)	AUCAll	Min AUC
**No CST**	**α = 0**	0.986	0.919 (−0.067)	0.928 (−0.058)	0.905 (−0.081)	0.984 (−0.002)	0.974 (−0.012)	0.953	**0.905**
**CST1**	**α = 1, DC = 1**	0.986	0.901 (−0.085)	0.936 (−0.050)	0.931 (−0.055)	0.983 (−0.003)	0.973 (−0.013)	0.950	**0.901**
**CST2**	**α = 1, DC = 2**	0.985	0.921 (−0.064)	0.957 (−0.028)	0.962 (−0.023)	0.984 (−0.001)	0.977 (−0.008)	0.963	**0.921**
**CST3**	**α = 1, DC = 3**	0.978	0.940 (−0.038)	0.968 (−0.010)	0.956 (−0.022)	0.975 (−0.003)	0.975 (−0.003)	0.959	**0.940**
**CST4**	**α = 1, DC = 4**	0.987	0.933 (−0.054)	0.958 (−0.029)	0.954 (−0.033)	0.985 (−0.002)	0.980 (−0.007)	0.967	**0.933**
**CST5**	**α = 2, DC = 2**	0.986	0.920 (−0.066)	0.949 (−0.037)	0.954 (−0.032)	0.984 (−0.002)	0.974 (−0.012)	0.960	0.920
CST6	α = 10, DC = 2	0.989	0.918 (−0.071)	0.964 (−0.025)	0.961 (−0.028)	0.986 (−0.003)	0.983 (−0.006)	0.970	0.918
CST7	α = 100, DC = 2	0.980	0.867 (−0.113)	0.927 (−0.053)	0.921 (−0.059)	0.975 (−0.005)	0.960 (−0.020)	0.942	0.867
DA1	DC = 1	0.987	0.916 (−0.071)	0.952 (−0.035)	0.940 (−0.047)	0.985 (−0.002)	0.978 (−0.009)	0.961	0.916
DA2	DC = 2	0.839	0.921 (0.082)	0.898 (0.059)	0.919 (0.080)	0.848 (0.009)	0.852 (0.013)	0.857	0.848
DA2_v2	DC = 2*	0.984	0.915 (−0.069)	0.948 (−0.036)	0.935 (−0.049)	0.982 (−0.002)	0.973 (−0.011)	0.957 / 0.915	0.915
DA3	DC = 3*	0.977	0.930 (−0.047)	0.940 (−0.037)	0.932 (−0.045)	0.972 (−0.005)	0.965 (−0.012)	0.944	0.930
DA4	DC = 4*	0.979	0.925 (−0.054)	0.948 (−0.031)	0.941 (−0.038)	0.976 (−0.003)	0.970 (−0.009)	0.950	0.925

Values in parenthesis correspond to AUCtestset − AUCbase. Cells marked in green represent the best performing (i.e., highest AUC) model for each test set, respectively. CST models had the highest AUC in all test sets. *Within the DCs used to train DA2_v2, DA3 and DA4, the *σ_K_* was set to $\frac{\sigma }{255}$ . Overall robustness is measured as the minimum performance delivered over all distortions.

Regarding the test sets with different IHC stains, performance relative to *P16-HT-baseline* decreased by 0.002 (AUC_CD3_ = 0.984) and 0.012 (AUC_CD8_ = 0.974), as compared to the performance in *P16-HT-baseline*. Interestingly, the CD3 and CD8 biomarkers do not stain the neoplastic epithelial tissue like p16, but model “No CST” was still able to correctly classify neoplastic epithelium tiles without p16 expression, despite never having seen it before (remembering that model “No CST” was trained exclusively with p16 WSIs, where neoplastic epithelium is stained in brown as shown in Figure 1). This shows that, even though there was a decrease in performance, model “No CST” was able to generalize through normal DL model training.

Regarding the segmentation on WSIs from different scanners, a greater decrease in performance was observed for model “No CST.” AUC for the *P16-XR* and *P16-S360* decreased in 0.058 and 0.081, respectively (AUCXR = 0.928, AUCS360 = 0.905) when compared to the performance on the P16-HT-baseline. Performance on the P16-HT-oof test set (i.e., scanned out of focus) was also significantly lower than on the baseline test set (rAUCoof = −0.067).

**Figure 4:**
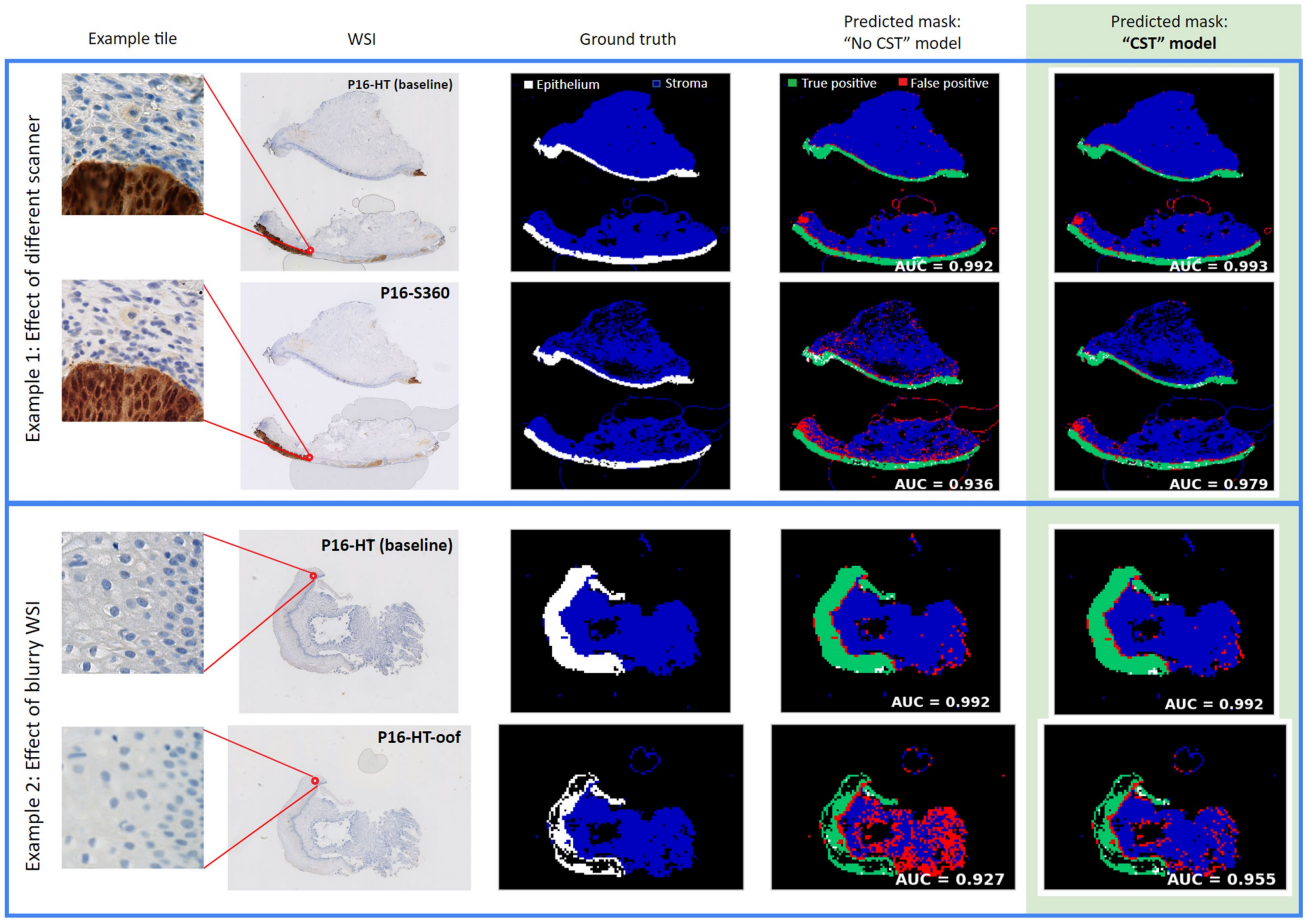
Examples of epithelium segmentation with and without CST. “No CST” model has a lower performance than CST models on WSIs from a different domain than the one used for training. CST models also outperform on WSIs from the same domain used for training.

The lowest relative performance was observed on the *P16-S360* test set, most likely caused by the noticeable difference in color and contrast between the NanoZoomer HT and the NanoZoomer S360. The low performance on the *P16-HT-oof* test set also shows that variability caused by scanning out-of-focus had a high impact in segmentation performance, even when using the same scanner and evaluating on the exact same slides. Two segmentation comparisons can be observed in Figure 4, where the column *Predicted mask: model “No CST”* shows the segmentation results for slides from the *P16-HT-baseline* as well as for *P16-S360* and *P16-HT-oof.* In both comparisons, the decrease in performance is shown as a clear increase in the number of False Positive tiles.

This experiment allowed us to quantify the effect that different scanners and scan quality (e.g., blurriness due to poor focus) had on DL model performance. Even though the exact same glass slides were used to build the baseline test set and the remaining scanner-distorted test sets (*P16-S360, P16-HT-oof* and *P16-XR*), the segmentation performance of model “No CST” on these was noticeably lower for the latter.

### Segmentation performance with CST and data augmentation

After obtaining the reference performances of model “No CST,” the next step was to evaluate models with CST to determine if there is an improvement in performance and, therefore, a higher robustness to distortions associated with real-world image variability. CST models were trained using the training set *P16-HT Train* as well; however, different values of α (1, 2, 10, 100) and different DCs (1, 2, 3, 4) were used. Additionally, five models were trained using data augmentation. Models DA1-DA4 were trained using DC1-4, respectively, to enable comparability between CST and traditional data augmentation. However, as described in Table 2, a second version of DA2 was trained (DA2_v2), and the value of σK for the training process of models DA2_v2, DA3 and DA4 was reduced to $\frac{\sigma }{255}$ to reduce the negative impact of a high level of blurring.

**Figure 5:**
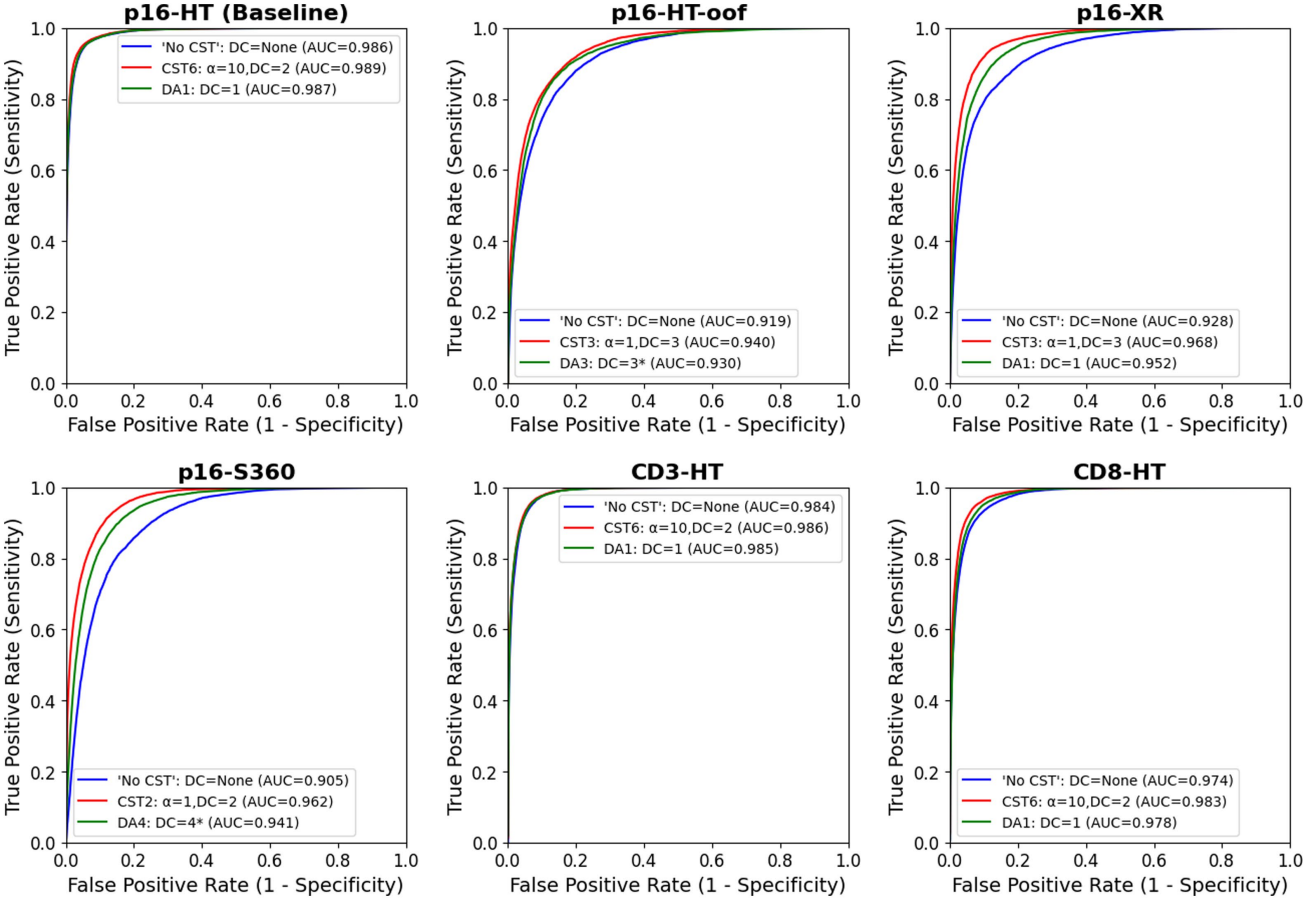
ROC curve of the “No CST” (blue), best CST and best DA models for each test set. In all test sets, CST outperformed “No CST” and DA, including the baseline testing set, P16-HT-baseline. In all testing sets, the improvement is statistically significant based on DeLong’s paired test for correlated ROC curves (*p* < 1.40e − 4). The correlation tests between all models are available in Supplementary Tables 2–8 for each test set.

Figure 5 shows the ROC curves and the AUC of the “No CST” model (blue) and the best CST model (red) for each test set. At a first glance, it can be observed that the best CST model outperformed model “No CST” in all test sets. The difference in AUC is statistically significant in all cases, based on the DeLong’s paired test for correlated ROC curves. The *p*-values of the correlation test between all models are available in Supplementary Tables 2–8 for each test set. Greater improvements were observed in the test sets with scanner variations, P16-HT-oof (0.919 to 0.940), P16-XR (0.928 to 0.968) and P16-S360 (0.905 to 0.962). Improvements were also observed, to a lower degree, on the test sets with IHC-based variability: CD3-HT (0.984 to 0.986), CD8-HT (0.974 to 0.983). Results displayed in Table 4 show that the best performing model (i.e., highest AUC) was a CST model in all test sets (highlighted in green).

Within the CST models, CST7 (α = 100, DC = 2) showed a low performance consistently in all test sets. This suggests that an excessively high value of α may have a counterproductive effect during model training, leading the model to prioritize robustness over performance. On the other hand, the DC did not seem to follow that same pattern. The model with the highest DC, CST4 (α = 1, DC = 4), had a high performance consistently in all test sets. However, the model with the highest overall AUC was CST6 (α = 10, DC = 2), with an AUC of 0.970 on the cumulated test set.

In terms of robustness, with the exception of model CST7, the least robust model (i.e., the model with the lowest rAUC_test_set_) was consistently the model “No CST” in all test sets. All CST models presented a higher robustness than model “No CST,” with the exception of the previously mentioned CST7 on the *P16-XR* test set. rAUC_test_set_ ranged from −0.002 to −0.081 for model “No CST,” while the best CST models ranged between −0.003 and −0.038.

**Figure 6:**
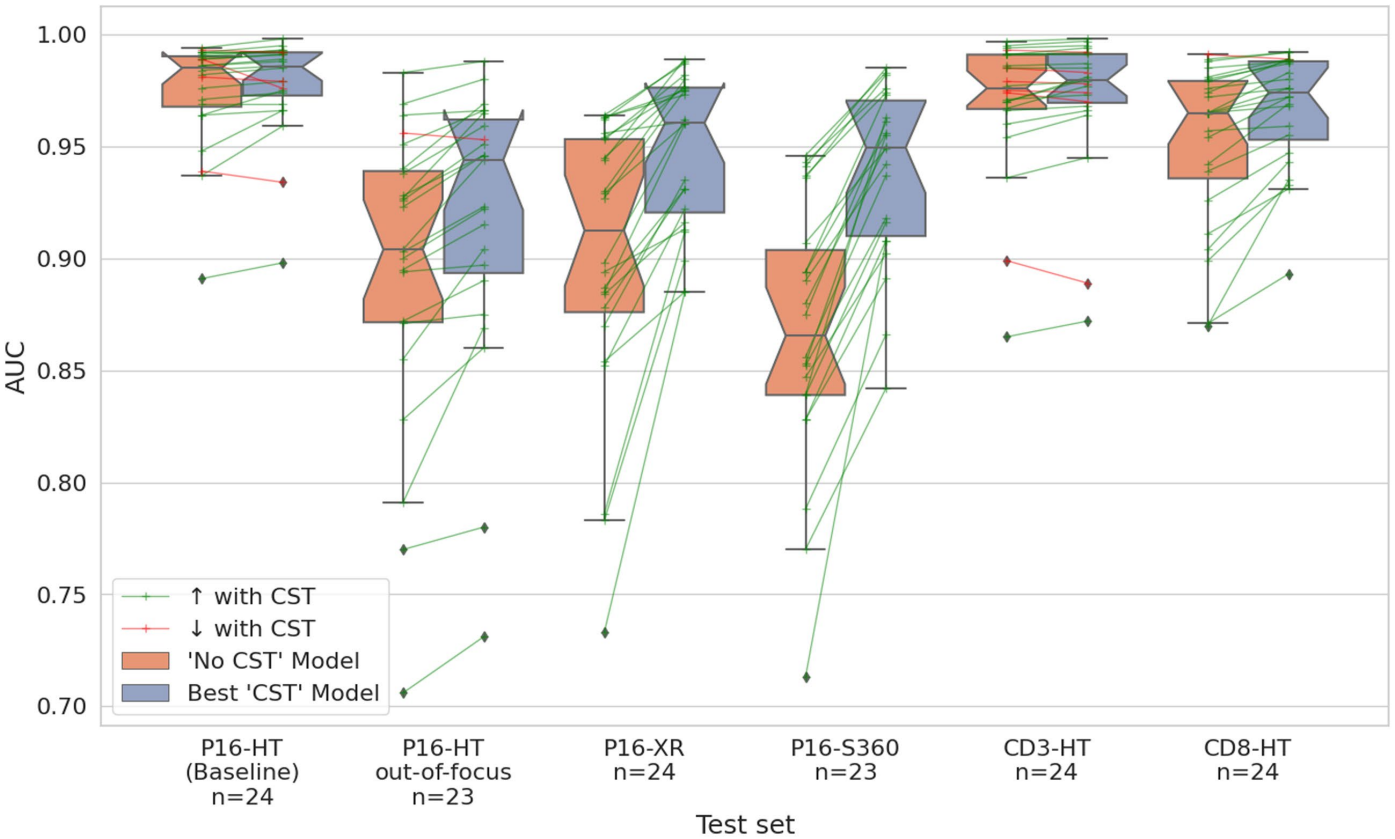
Distribution of the AUC per WSI on all test sets. Orange boxplots correspond to the results of the “No CST” model. Blue boxplots correspond to the results of the best CST model for each respective test set. The green arrow lines show WSIs where the model with CST outperformed the “No CST” model. The red arrow lines show WSIs where the opposite occurs.

From a WSI-wise perspective, Figure 6 shows the distribution of the AUC calculated for each individual WSI. The green lines represent WSIs where the AUC increased with CST over model “No CST,” while the red lines represent the opposite. CST models improved their AUC compared to “No CST” in 124 of the 142 WSIs with an improvement ranging between 0.001 and 0.195, while 5 WSIs had no improvement and 13 WSIs had a decrease ranging between −0.013 and −0.001. Supplementary Figures 1–8 comparing the performance of all models is available in Supplementary material.

The models with data augmentation showed improved relative predictive performance, as well as an improvement in relative robustness as compared with the “No CST” model. However, the best CST models outperformed the best DA models across all test sets ([0.002, 0.021], *p* < 1e − 6).

A significant performance decrease was observed across almost all datasets for model DA2, especially on WSIs coming from the HT scanner (AUC between 0.839 and 0.921), suggesting that the inclusion of blur in the distortion layer might have caused overfitting. The decision was made to reduce the value of σK for the training process of DA2_v2, DA3 and DA4. The performance of these three models on images from the same scanner (P16-HT, CD3-HT and CD8-HT) was lower than the performance of DA1, suggesting a possible underfitting associated with the higher color, contrast and brightness distortions from DC3 and DC4, as well as blur on the three models. The training process with CST seemed more tolerant to high levels of distortion during training. CST3 and CST4 show a good performance on blurry images, as well as images from different scanners, even with high distortion levels for all image properties.

## Discussion

Training robust CNNs is a challenge in all fields of application of DL. Humans are able to bypass sources of image variability that actually confuse DL models (12). Gaussian noise or salt and pepper patterns, slight color perturbations, blur and other distortions do not prevent people from recognizing objects, but neural networks may struggle even if these perturbations are visually unnoticeable (12, 26). To the authors’ knowledge, currently there is no research on the robustness to variability caused by different scanners and IHC for cervical WSI segmentation tasks. Additionally, no studies involving the use of CST were found to address this issue.

In the context of digital pathology, different methods have been applied to improve DL robustness. Domain adaptation has been used to obtain stain invariant features between multi-sourced WSIs during CNN training (27, 28), although results appear not to be conclusive either due to the CNN classification performance or to the size of the validation set. Data augmentation has also been applied to increase robustness, by adding images with color space or pixel level distortions (29), even in multi-center and multi-organ experimental settings (30). Data augmentation is easy to implement and can help increase robustness to image variability. Nonetheless, the challenge of addressing the high heterogeneity caused by different sample preparations, scanners and scanning conditions still persists. It has also been observed that the use of data augmentation with distorted images may cause underfitting (16).

Stain color normalization has also been used to address WSI heterogeneity (15, 30–33). While the previously described methods attempt to make DL models robust to image variability within the training process, stain normalization is intended as a preprocessing step to reduce image variability before the classification or segmentation task. Sparse autoencoders have been applied to reduce staining heterogeneity, reducing the difference between “template” and “distorted” images (15). Multi-scale feature extraction has also been used to eliminate color deviations from a base image set (32).

Recently, contrastive learning has gained popularity due to its remarkable performance in classification tasks by means of unsupervised training (17, 34–36). This constitutes a novel application of a relatively old concept, where the similarity between feature embeddings is calculated to train networks to recognize similar and dissimilar images (37). Feature embedding and softmax output contrasting though similarity-based loss functions has also been used to increase robustness to different types of distortions, primarily in the context of object classification (38). Before the term was coined as such, Contrastive Learning-based methods have been applied to increase robustness to compression loss, downscaling and rescaling, random cropping and gaussian noise as sources of image variability (16, 23). Although these methods have only been tested on artificially distorted test sets, they contributed toward the design of this work. Additionally, to the authors’ knowledge these methods have not been evaluated directly to address robustness in digital pathology, nor have these been validated in a real world scenario with heterogeneous WSIs.

In the field of digital pathology, using DL models that are robust to real-world image distortions can enable large-scale research with WSIs from different sources and with different preparations. This was, as a matter of fact, our major motivation for this work. Within our experimental setup, we sized the potential effect of real-world image distortions in the automatic segmentation of epithelium in cervical WSIs, and we evaluated if CST can increase DL model robustness to such distortions. For this purpose, we generated WSI datasets using different scanner models and samples with different IHC, which we used to evaluate segmentation performance of DL models trained with and without CST.

In general, epithelium was accurately segmented on WSIs from the same scanner used for training (i.e., Hamamatsu-HT). Interestingly, although the DL models were trained exclusively with p16-stained tissue, neoplastic epithelium was also correctly classified for WSIs stained for CD3/CD8. Similarly, the presence of stained CD3+ and CD8+ T cells in the stroma did not affect the correct classification of tissue as stroma. Overall, DL models trained with CST still had a higher performance on these test sets, including the set from the same domain as the training set (i.e., p16-stained from NanoZoomer-HT). A possible explanation is that there may be visually imperceptible “signatures” for each scanner, caused by the combination of hardware and software used in the generation of the images. Different lenses, lights, z-plane calculation algorithms and focal point selection algorithms are just some of these. There are already studies that show the poor generalization between images from different institutions. These even show that CNNs can detect the institutions where images were generated, which further validates the theory of a “signature” (39).

Regarding segmentation on WSIs from different scanner models (i.e., NanoZoomer-XR and NanoZoomer-S360), results showed a significant lack of robustness from the “No CST” model. While it correctly segmented WSIs from the same domain as the training set, segmentation performance decreased significantly on WSIs from different scanner models, even though these WSIs were generated using the exact same slides. This poor segmentation of WSIs from different scanners further supports the idea of a possible scanner “signature.” Similarly, segmentation on WSIs scanned out-of-focus suffered a significant decrease in performance, as expected due to the loss of information caused by the blurriness.

In comparison, CST proved to be effective in increasing robustness to real-world image distortions, given the experimental setup used for this work. The best CST model outperformed the “No CST” model in all test sets without exception, thus validating this method as a potential first line of defense to image variability. Performance of CST varied depending on the weight of the stability component (i.e., *α*) and the magnitude of the distortions (i.e., DC) used in the setup for this work. Our results show that increasing the weight of the stability component *α* improves DL model performance up to a certain level, but performance decreases after that level. This makes sense intuitively: for example, a loss function with α=∞ will force the DL model to maximize stability while completely disregarding the accurate prediction of the input class. On the other hand, increasing the *DC* (i.e., the strength of the distortion), seems to further improve the predictive performance.

When compared with data augmentation, CST proved to be more effective in general for the selected ɑ and DC values. Results suggest that using strong distortions may lead to model underfitting when training with data augmentation, as observed with blur and on a lower degree with color, brightness and contrast. This may be due to the introduction of unrealistic variations in the images, which do not reflect the true distribution of the data, leading models to learn these variations instead of the relevant image patterns. In this context, the performance of data augmented models was consistent with the literature regarding the subject. The fact that CST uses the original images during the training process may be attenuating the effect of these high distortions, therefore reducing the risk of underfitting.

The main limitation of CST is that it doubles the computing time during training, since the input is doubled on every training iteration (16). However, each distorted image has a random variation of its properties, so the DL model learns to stabilize from multiple random distortions, as opposed to statically augmented datasets. For further research in robustness, we would like to increase the number of test slides and evaluate CST on slides from different sources (e.g., new scanners and new scanning conditions), as well as evaluating the effect of distortions on single properties to gain a deeper understanding of the effect these hyper-parameters have on robustness and overall performance. Another limitation of this method is the optimization of ɑ and DC. Here, this was done through trial and error in an approach similar to grid search, which requires training and evaluating multiple models. A potential improvement for CST may be to incorporate the optimization of ɑ and DC during training, as additional training parameters.

The clinical motivation behind this work is to develop automated, robust methods to generate quantitative metrics that support the diagnosis and risk stratification of CIN and cervical cancer. This work specifically focused on the automated detection of epithelial tissue using deep learning. Further work will address the extraction of quantitative metrics from WSIs of cervical histology, including metrics to quantify the color and morphological patterns associated with IHC stains. For this purpose, challenges associated with scanner and IHC-based image variability will need to be addressed as well. The focus of this work was on biomarkers for p16, CD3, and CD8 given their use in the diagnosis, prognosis and risk stratification of cervical cancer and other forms of squamous carcinoma.

Immune cells have been reported as biomarkers to predict patient outcome and response to treatment (40). T cells have shown higher density in stromal regions where there is inflammation of tissue caused by the presence of tumors. The presence of CD3+ and CD8+ T cells and their relevance for the prognosis of colorectal cancer has been known principally since the 1980s (41, 42). But due to their dispersed appearance only the advent of digital pathology allowed their exact quantification serving as a basis for prognosis and subsequently also prediction of patient response (43).

More recently, regarding cervical cancer, immune cell analysis has also been used to evaluate patient response to experimental treatments for cervical cancer, as well as to analyze immune response to chemotherapy (44, 45). In the case of CIN, immune cell analysis was performed on a small cohort to evaluate the relation between immune responses and CIN grade progression (46). Examples like these suggest that immune cell analysis in cervical cancer is currently an open field, with potential to assist in the research for new treatments. It could also be a useful method to stratify patients based on their cancer type, to evaluate response to treatment and to predict patient outcome. The use of IHC enables the visualization of immune cells, such as CD3+ and CD8+ T cells.

The automatic quantification of multiple IHC stains on different sections of the same tissue block may provide insight into the underlying relations between the biological events that trigger their respective biomarker expressions. The relation between immune cell response and epithelial neoplastic tissue can be monitored to stratify risk, evaluate response to treatment and increase prognostic accuracy. Additionally, a shift from a qualitative to a quantitative assessment of biomarker expression may assist in reducing the high interobserver variability that is currently observed in CIN diagnosis (47–49). Reproducible quantitative metrics can be generated by using automated image analysis tools, making diagnoses explainable and easier to share within interconsultation between pathologists.

## Data availability statement

The data analyzed in this study is subject to the following licenses/restrictions: The availability of the data used for this work is regulated by the DCEG-NCI. Requests to access these datasets should be directed to wentzenn@mail.nih.gov.

## Ethics statement

The images in this study were generated from SUCCEED. SUCCEED was a cross-sectional study of women 18 years of age or older with an abnormal Pap smear who were referred to colposcopy or treatment at the University of Oklahoma (OUHSC) between 2003 and 2011. Written informed consent was obtained from all women enrolled in the study, and Institutional Review Board approval was provided by OUHSC and the US National Cancer Institute (NCI).

## Author contributions

FM and NG conceived and designed the study. LB, AKr, AKe, AT, MC, and NW collected and prepared the biopsy slides and collected the clinical data. FM developed the code and analyzed the data. FM and BL wrote the manuscript. PS, MC, SH, NW, and NG revised the manuscript. BL, SH, and NG supervised the manuscript. All authors contributed to the article and approved the submitted version.

## Funding

SH was supported by ANID (ICN09_015, ICM P09-015-F, and FONDECYT 1211988), RED 21994 MINEDUC, and DAAD 57519605. FM, BL, AKe, and NG were supported by the “Data Science Region Lower Saxony” Grant and the BMBF project “CancerScout.” FM, BL, AKe, LB, and NG were supported by the Steinbeis Center for Medical Systems Biology (STCMED) providing research analysis services.

## Conflict of interest

The authors declare that the research was conducted in the absence of any commercial or financial relationships that could be construed as a potential conflict of interest.

## Publisher’s note

All claims expressed in this article are solely those of the authors and do not necessarily represent those of their affiliated organizations, or those of the publisher, the editors and the reviewers. Any product that may be evaluated in this article, or claim that may be made by its manufacturer, is not guaranteed or endorsed by the publisher.

## Abbreviations

CNN: Convolutional neural network
CST: CNN stability training
WSI(s): Whole slide image(s)
DL: Deep learning
CIN: Cervical intraepithelial neoplasia
HT: Hamamatsu NanoZoomer HT
S360: Hamamatsu NanoZoomer S360
XR: Hamamatsu NanoZoomer XR
AUC: Area under the ROC Curve
DC: Distortion combination
IHC: Immunohistochemistry
