# Supplementary material for: CNN stability training improves robustness to scanner and IHC-based image variability for epithelium segmentation in cervical histology
Source: Front Med (Lausanne). 2023 Jul 5;10:1173616. doi: 10.3389/fmed.2023.1173616 (PMC10354251; doi:10.3389/fmed.2023.1173616)
Supplement: Supplementary file 1 [file Data_Sheet_1.docx]

***Supplementary Material***

**CNN Stability Training improves robustness to scanner and IHC-based image variability for epithelium segmentation in cervical histology**

**Felipe Miranda Ruiz^1,3^, Bernd Lahrmann^1,3^, Liam Bartels^2,3^, Alexandra Krauthoff^2,3^, Andreas Keil^1,3^, Amy S. Tao^4^, Philipp Ströbel^1^, Megan A. Clarke^4^ , Steffen Härtel^5^, Nicolas Wentzensen^4^, Niels Grabe^1,2,3*^**

^1^ Institute of Pathology, University Medical Center Göttingen UMG, Göttingen, Germany

^2^ Medical Oncology Department, National Center for Tumor Diseases (NCT), Heidelberg, Germany

^3^ Hamamatsu Tissue Imaging and Analysis Center (TIGA), BIOQUANT Center, Heidelberg, University, Heidelberg, Germany

^4^ Division of Cancer Epidemiology and Genetics, US National Cancer Institute (NCI), Bethesda, Maryland, United States

^5^ Laboratory for Scientific Image Processing (SCIAN-Lab), Center of Medical Informatics (CIMT), Biomedical Neuroscience Institute (BNI), ICBM, Medical Faculty, University of Chile, Santiago, Chile.

*** Correspondence:**Niels Grabe, PhD
niels.grabe@med.uni-goettingen.de

## Supplementary Figures

**
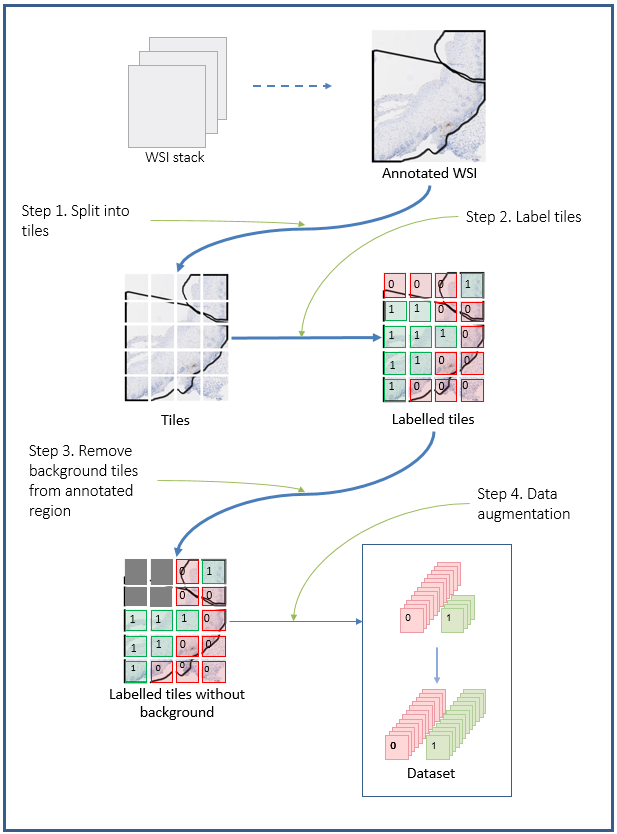
**

**Supplementary figure 1.** Method for training dataset curation. Step 1. The original WSI is split into tiles. Step 2. Tiles are labeled as 1 (“epithelium”) or 0 (“non-epithelium”) depending on whether these are inside or outside the annotated region, respectively. Partially labeled tiles are labeled as 0. Step 3. Tiles corresponding to background are removed from the set. Step 4. Data augmentation is applied to the smallest class to solve class imbalance.


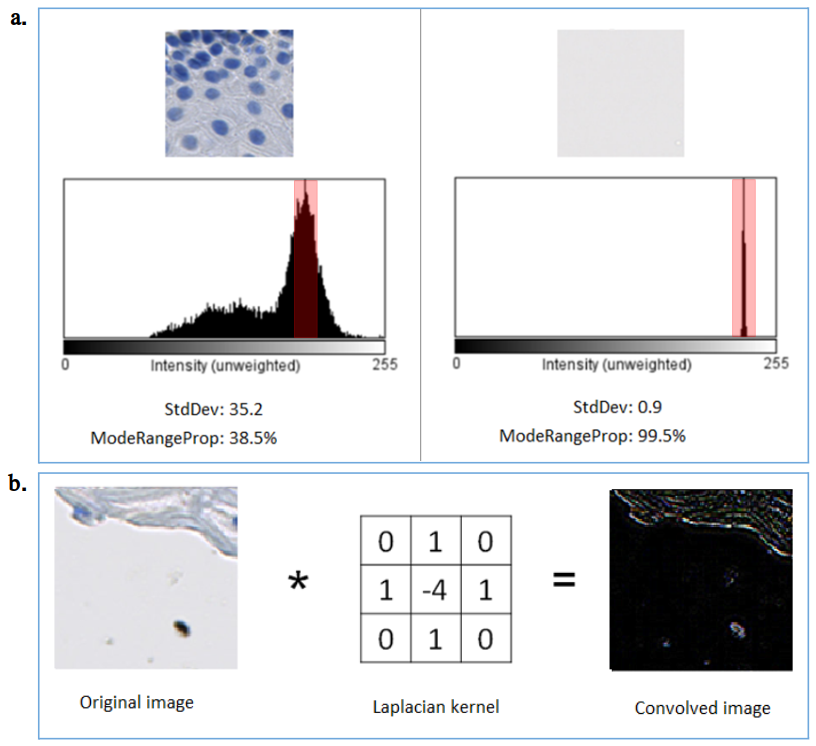


**Supplementary Figure 2.** Two-step process to identify background tiles. *(a)* Example of a histogram analysis for two tiles. The standard deviation (StdDev) is calculated as the distribution of the pixel color in the image and the proportion of pixels within a range from the mode (ModeRangeProp) is calculated as the proportion of pixels within the red region over the total number of pixels in the image. *(b)* Example of the convolution of an image with a laplacian kernel. The resulting convolved image highlights the edges of the original image.


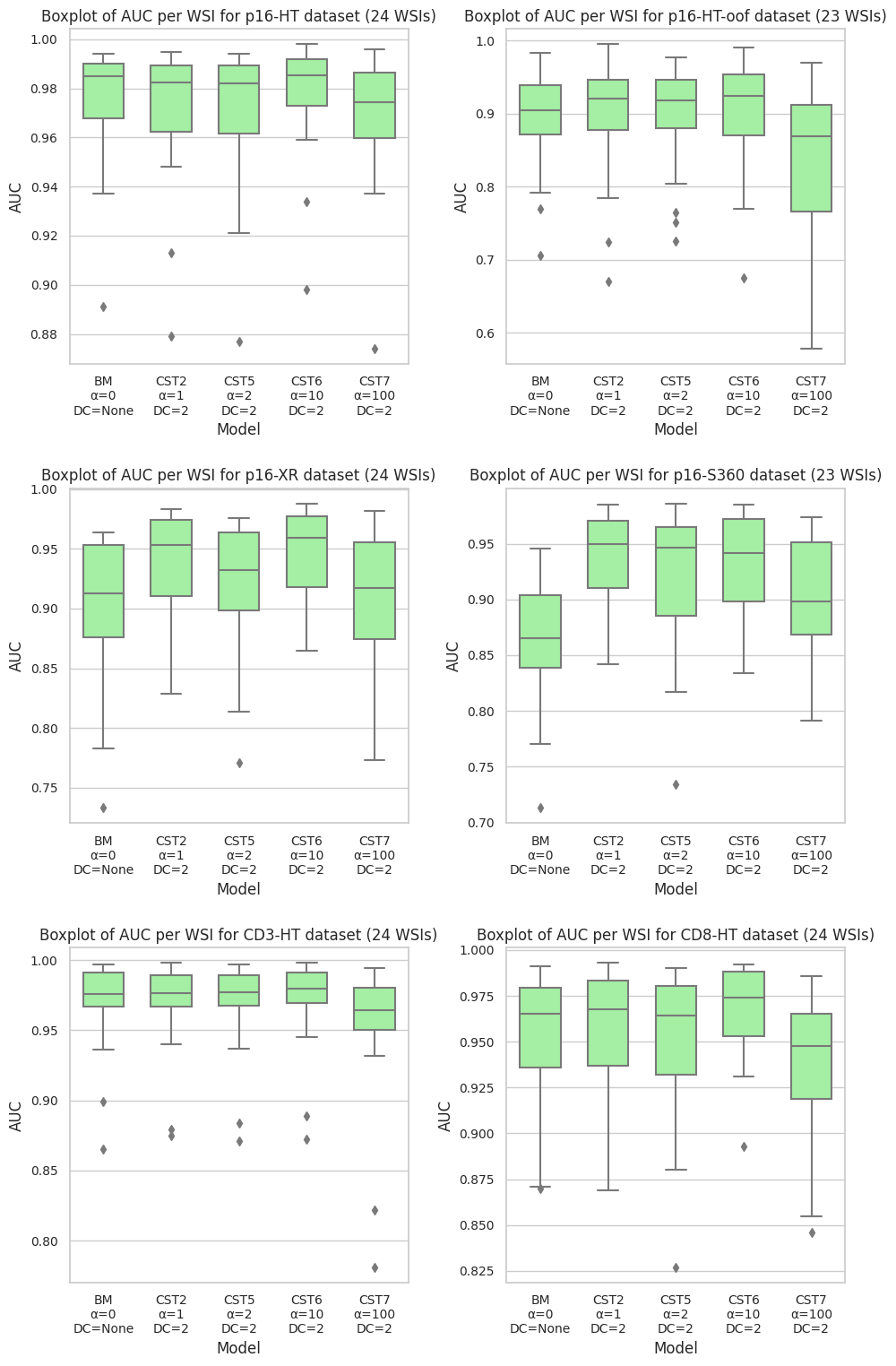


**Supplementary Figure 3.** Boxplot of AUC per WSI for each test set, comparing the performance of CST models with varying values for parameter α. BM is the model without CST.


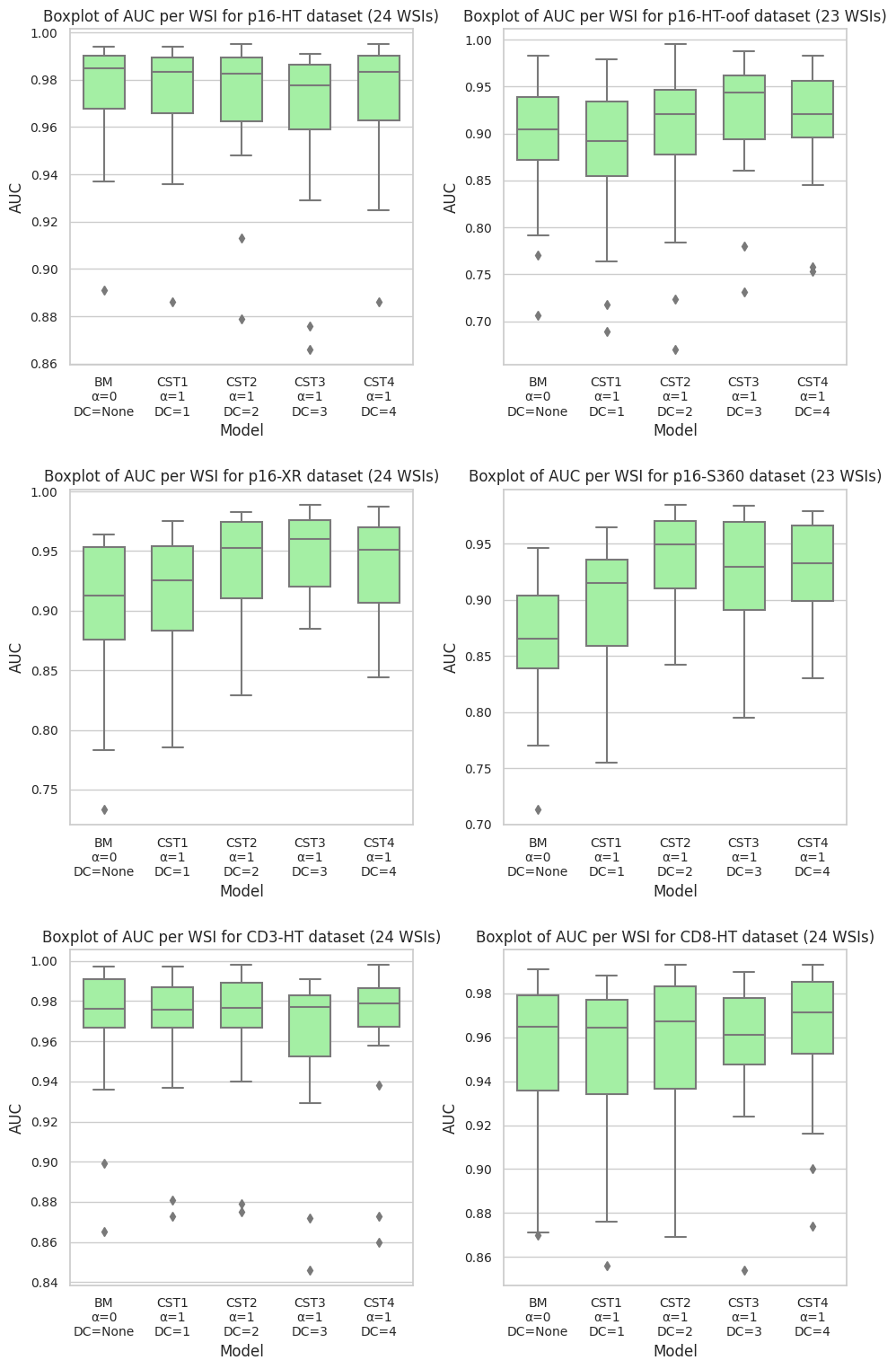


**Supplementary figure 4.** Boxplot of AUC per WSI for each test set, comparing the performance of CST models with varying values for parameter DC. BM is the model without CST.


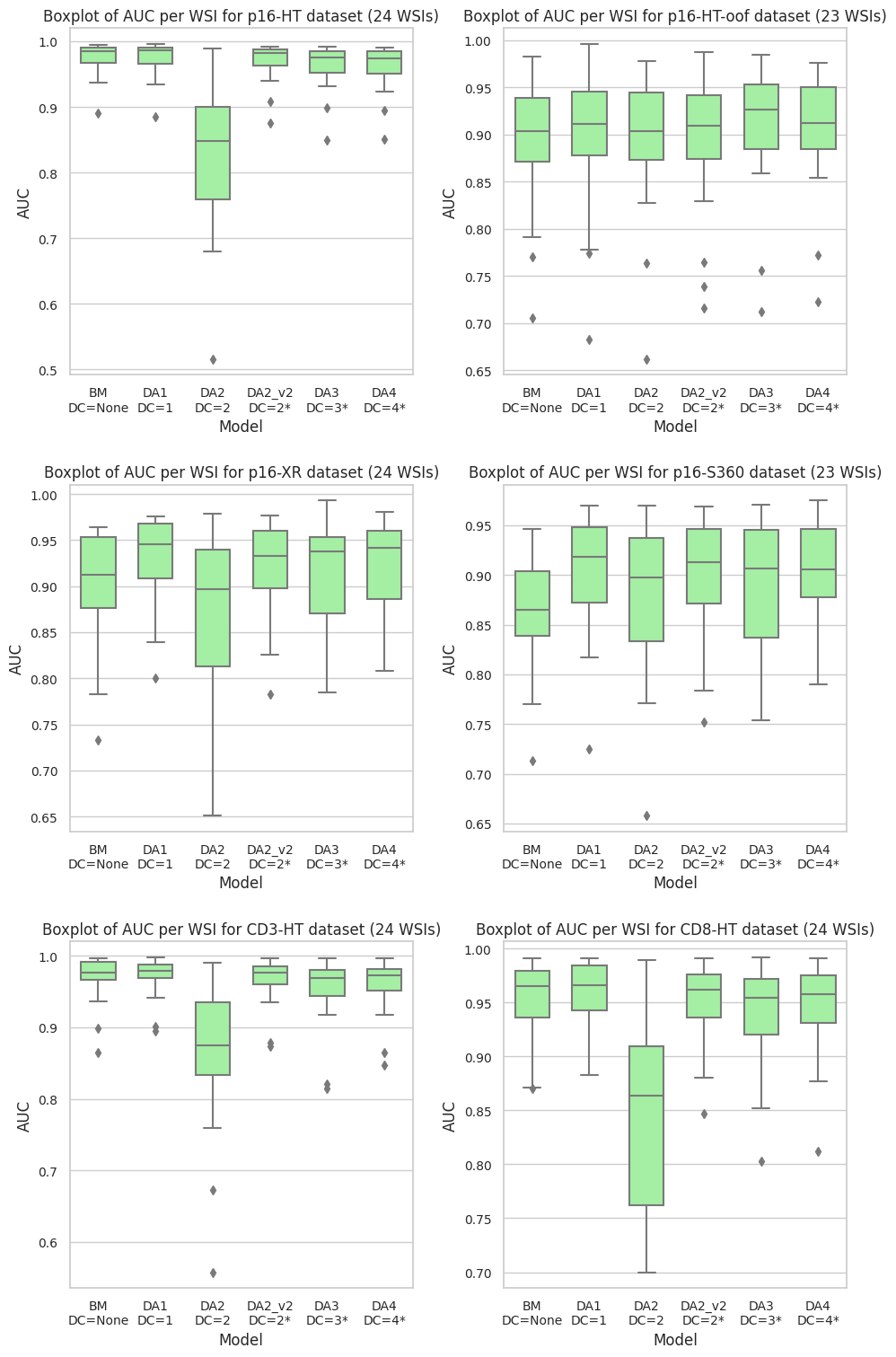


**Supplementary figure 5.** Boxplot of AUC per WSI for each test set, comparing the performance of DA models with varying values for parameter DC. BM is the model without DA. *The value of σ in the Blur image property of DA2_v2, DA3 and DA4 was set to $\frac{\sigma}{255}$.

**
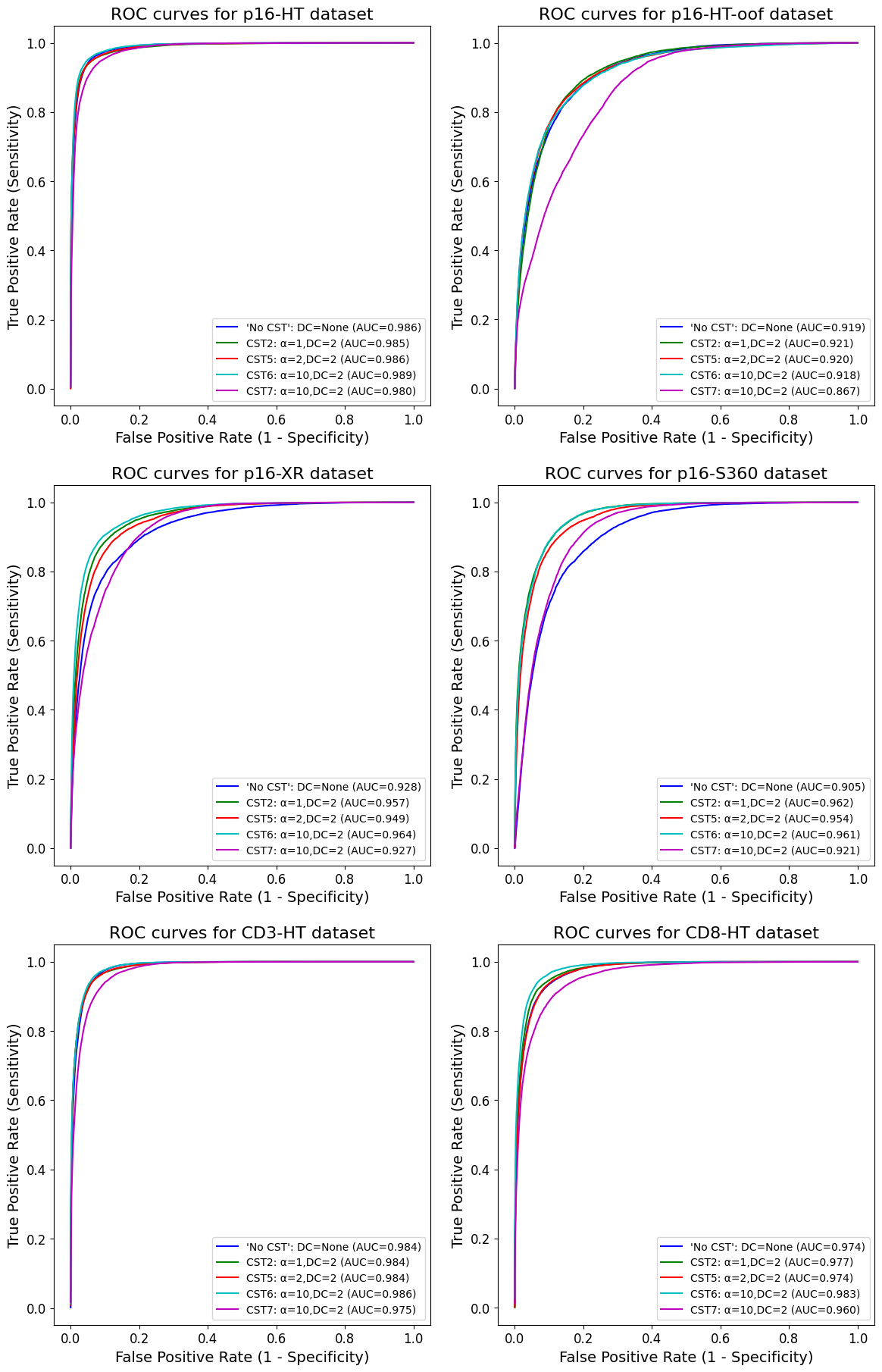
**

**Supplementary figure 6.** Tile-based comparative AUC results for CST models with varying values for parameter α.


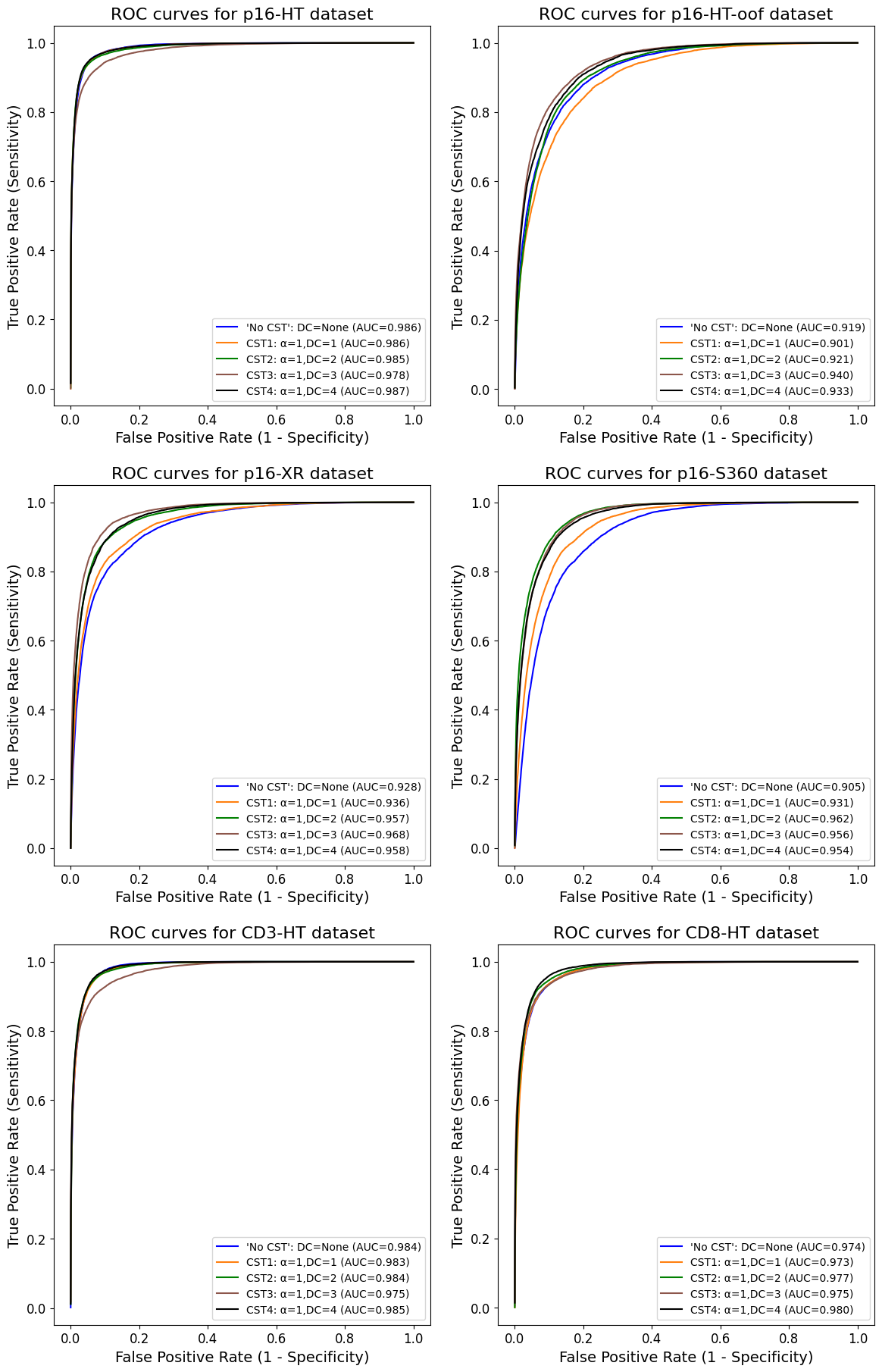


**Supplementary figure 7.** Tile-based comparative AUC results for CST models with varying values for parameter DC.


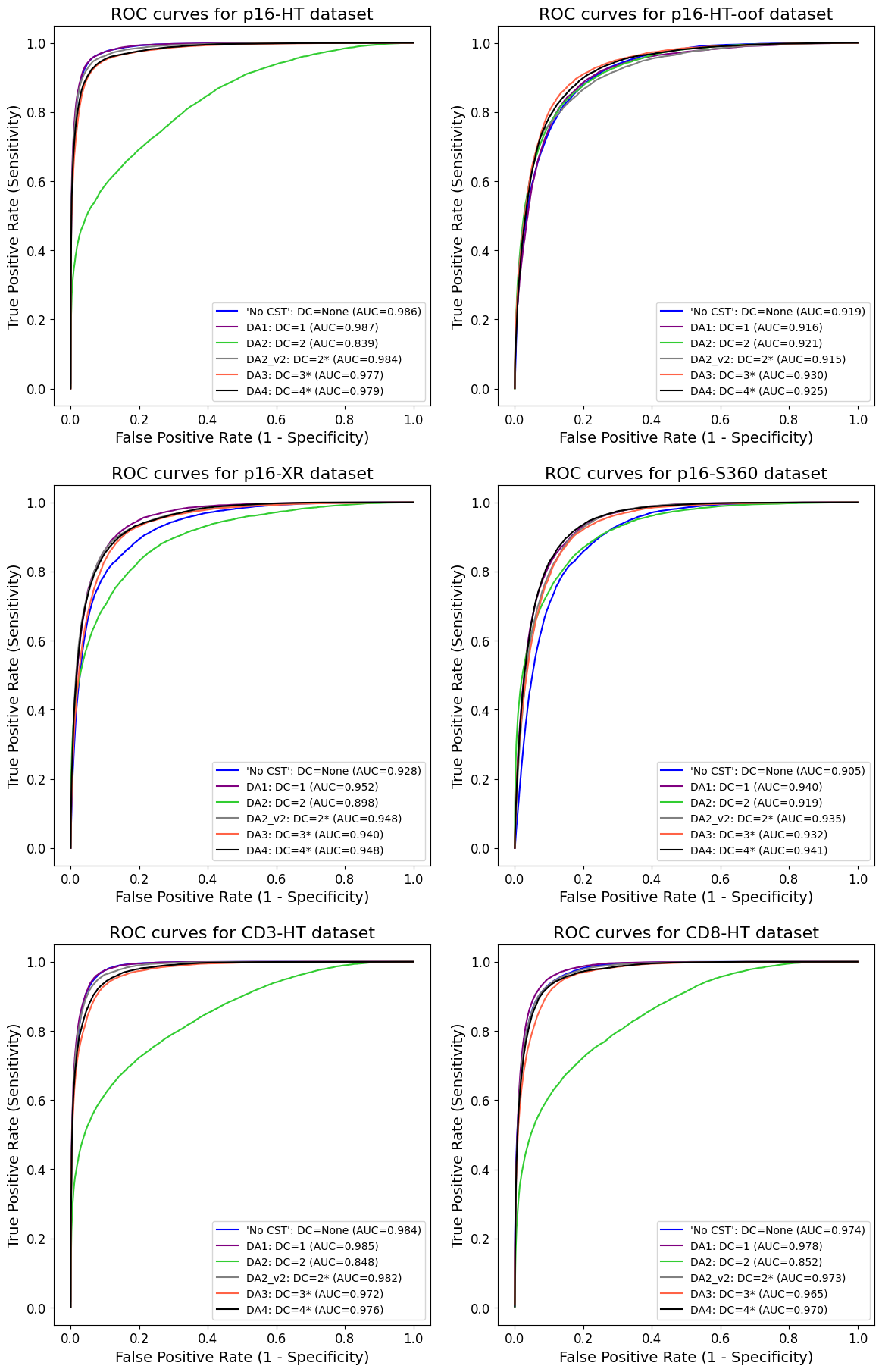


**Supplementary figure 8.** Tile-based comparative AUC results for CST models with varying values for parameter DC. *The value of σ in the Blur image property of DA2_v2, DA3 and DA4 was set to $\frac{\sigma}{255}$.

## 1.2. Supplementary tables

**Supplementary table 1.** AUC of all submodels used to create BM, CST and DA ensemble models.

| **Model** | **Parameters** | **P16-HT (baseline)** | **P16-HT-oof** | **P16-XR** | **P16-S360** | **CD3-HT** | **CD8-HT** | **All test sets** |
| --- | --- | --- | --- | --- | --- | --- | --- | --- |
| No CST_1 | α=0 | 0.983 | 0.905 | 0.898 | 0.878 | 0.981 | 0.966 | 0.941 |
| No CST_2 | α=0 | 0.986 | 0.905 | 0.929 | 0.908 | 0.984 | 0.973 | 0.95 |
| No CST_3 | α=0 | 0.984 | 0.929 | 0.933 | 0.9 | 0.981 | 0.972 | 0.954 |
| CST1_1 | α=1, DC=1 | 0.985 | 0.894 | 0.925 | 0.921 | 0.982 | 0.97 | 0.944 |
| CST1_2 | α=1, DC=1 | 0.983 | 0.897 | 0.92 | 0.917 | 0.98 | 0.967 | 0.939 |
| CST1_3 | α=1, DC=1 | 0.986 | 0.913 | 0.941 | 0.932 | 0.983 | 0.973 | 0.953 |
| CST2_1 | α=1, DC=2 | 0.985 | 0.914 | 0.955 | 0.953 | 0.983 | 0.978 | 0.963 |
| CST2_2 | α=1, DC=2 | 0.985 | 0.921 | 0.948 | 0.953 | 0.984 | 0.974 | 0.96 |
| CST2_3 | α=1, DC=2 | 0.982 | 0.908 | 0.954 | 0.963 | 0.981 | 0.971 | 0.953 |
| CST3_1 | α=1, DC=3 | 0.977 | 0.935 | 0.967 | 0.955 | 0.973 | 0.973 | 0.958 |
| CST3_2 | α=1, DC=3 | 0.98 | 0.936 | 0.966 | 0.954 | 0.977 | 0.974 | 0.958 |
| CST3_3 | α=1, DC=3 | 0.97 | 0.941 | 0.962 | 0.949 | 0.964 | 0.968 | 0.953 |
| CST4_1 | α=1, DC=4 | 0.986 | 0.928 | 0.956 | 0.95 | 0.984 | 0.977 | 0.963 |
| CST4_2 | α=1, DC=4 | 0.985 | 0.925 | 0.956 | 0.95 | 0.984 | 0.98 | 0.965 |
| CST4_3 | α=1, DC=4 | 0.985 | 0.936 | 0.956 | 0.953 | 0.983 | 0.98 | 0.966 |
| CST5_1 | α=2, DC=2 | 0.981 | 0.907 | 0.93 | 0.925 | 0.979 | 0.962 | 0.945 |
| CST5_2 | α=2, DC=2 | 0.985 | 0.918 | 0.952 | 0.955 | 0.984 | 0.973 | 0.96 |
| CST5_3 | α=2, DC=2 | 0.986 | 0.921 | 0.946 | 0.953 | 0.985 | 0.974 | 0.961 |
| CST6_1 | α=10, DC=2 | 0.988 | 0.923 | 0.963 | 0.948 | 0.986 | 0.982 | 0.968 |
| CST6_2 | α=10, DC=2 | 0.988 | 0.903 | 0.959 | 0.963 | 0.985 | 0.981 | 0.967 |
| CST6_3 | α=10, DC=2 | 0.988 | 0.921 | 0.965 | 0.962 | 0.986 | 0.983 | 0.97 |
| CST7_1 | α=100, DC=2 | 0.979 | 0.859 | 0.919 | 0.907 | 0.972 | 0.952 | 0.934 |
| CST7_2 | α=100, DC=2 | 0.981 | 0.877 | 0.929 | 0.923 | 0.977 | 0.965 | 0.946 |
| CST7_3 | α=100, DC=2 | 0.978 | 0.86 | 0.928 | 0.924 | 0.972 | 0.956 | 0.94 |
| DA1_1 | DC=1 | 0.986 | 0.896 | 0.95 | 0.945 | 0.985 | 0.977 | 0.96 |
| DA1_2 | DC=1 | 0.981 | 0.919 | 0.939 | 0.903 | 0.98 | 0.967 | 0.946 |
| DA1_3 | DC=1 | 0.987 | 0.912 | 0.938 | 0.916 | 0.985 | 0.975 | 0.954 |
| DA2_1 | DC=2 | 0.789 | 0.903 | 0.838 | 0.87 | 0.795 | 0.797 | 0.813 |
| DA2_2 | DC=2 | 0.825 | 0.923 | 0.899 | 0.901 | 0.827 | 0.839 | 0.848 |
| DA2_3 | DC=2 | 0.852 | 0.917 | 0.904 | 0.928 | 0.871 | 0.866 | 0.867 |
| DA2_v2_1 | DC=2* | 0.979 | 0.9 | 0.936 | 0.919 | 0.977 | 0.966 | 0.947 |
| DA2_v2_2 | DC=2* | 0.979 | 0.925 | 0.943 | 0.929 | 0.976 | 0.967 | 0.949 |
| DA2_v2_3 | DC=2* | 0.985 | 0.915 | 0.947 | 0.928 | 0.984 | 0.973 | 0.954 |
| DA3_1 | DC=3* | 0.975 | 0.92 | 0.934 | 0.925 | 0.971 | 0.956 | 0.935 |
| DA3_2 | DC=3* | 0.977 | 0.925 | 0.939 | 0.93 | 0.971 | 0.962 | 0.941 |
| DA3_3 | DC=3* | 0.969 | 0.93 | 0.926 | 0.917 | 0.961 | 0.959 | 0.935 |
| DA4_1 | DC=4* | 0.978 | 0.918 | 0.945 | 0.937 | 0.973 | 0.971 | 0.95 |
| DA4_2 | DC=4* | 0.972 | 0.919 | 0.944 | 0.937 | 0.97 | 0.965 | 0.947 |
| DA4_3 | DC=4* | 0.97 | 0.907 | 0.916 | 0.89 | 0.97 | 0.942 | 0.924 |

**Supplementary table 2.** p-values for Delong paired AUC comparisons, p16-HT dataset

|  | No CST | CST1 | CST2 | CST3 | CST4 | CST5 | CST6 | CST7 | DA1 | DA2 | DA2_v2 | DA3 | DA4 |
| --- | --- | --- | --- | --- | --- | --- | --- | --- | --- | --- | --- | --- | --- |
| No CST | NaN | 4.2E-01 | 8.3E-06 | 5.2E-102 | 3.8E-01 | 4.8E-03 | 3.4E-21 | 3.7E-68 | 1.4E-04 | 0.0E+00 | 1.2E-23 | 6.0E-120 | 2.6E-84 |
| CST1 | 4.2E-01 | NaN | 3.4E-04 | 1.8E-95 | 1.1E-01 | 5.5E-02 | 2.6E-23 | 8.8E-60 | 2.0E-05 | 0.0E+00 | 1.2E-19 | 2.6E-120 | 2.5E-85 |
| CST2 | 8.3E-06 | 3.4E-04 | NaN | 2.4E-121 | 4.9E-09 | 3.8E-02 | 9.9E-38 | 9.6E-43 | 6.9E-17 | 0.0E+00 | 6.7E-11 | 4.2E-124 | 1.6E-91 |
| CST3 | 5.2E-102 | 1.8E-95 | 2.4E-121 | NaN | 1.1E-131 | 2.2E-113 | 1.6E-155 | 1.3E-05 | 8.7E-134 | 0.0E+00 | 9.4E-63 | 3.5E-03 | 1.2E-04 |
| CST4 | 3.8E-01 | 1.1E-01 | 4.9E-09 | 1.1E-131 | NaN | 7.1E-05 | 3.6E-23 | 2.0E-76 | 4.1E-03 | 0.0E+00 | 4.1E-31 | 5.8E-165 | 1.8E-120 |
| CST5 | 4.8E-03 | 5.5E-02 | 3.8E-02 | 2.2E-113 | 7.1E-05 | NaN | 7.2E-38 | 2.5E-52 | 2.9E-11 | 0.0E+00 | 2.4E-15 | 3.3E-127 | 3.8E-94 |
| CST6 | 3.4E-21 | 2.6E-23 | 9.9E-38 | 1.6E-155 | 3.6E-23 | 7.2E-38 | NaN | 1.7E-156 | 3.0E-12 | 0.0E+00 | 1.2E-67 | 1.4E-175 | 2.4E-138 |
| CST7 | 3.7E-68 | 8.8E-60 | 9.6E-43 | 1.3E-05 | 2.0E-76 | 2.5E-52 | 1.7E-156 | NaN | 1.3E-93 | 0.0E+00 | 7.8E-19 | 2.0E-09 | 7.5E-02 |
| DA1 | 1.4E-04 | 2.0E-05 | 6.9E-17 | 8.7E-134 | 4.1E-03 | 2.9E-11 | 3.0E-12 | 1.3E-93 | NaN | 0.0E+00 | 3.4E-45 | 1.7E-154 | 7.8E-120 |
| DA2 | 0.0E+00 | 0.0E+00 | 0.0E+00 | 0.0E+00 | 0.0E+00 | 0.0E+00 | 0.0E+00 | 0.0E+00 | 0.0E+00 | NaN | 0.0E+00 | 0.0E+00 | 0.0E+00 |
| DA2_v2 | 1.2E-23 | 1.2E-19 | 6.7E-11 | 9.4E-63 | 4.1E-31 | 2.4E-15 | 1.2E-67 | 7.8E-19 | 3.4E-45 | 0.0E+00 | NaN | 1.1E-85 | 5.6E-47 |
| DA3 | 6.0E-120 | 2.6E-120 | 4.2E-124 | 3.5E-03 | 5.8E-165 | 3.3E-127 | 1.4E-175 | 2.0E-09 | 1.7E-154 | 0.0E+00 | 1.1E-85 | NaN | 8.2E-12 |
| DA4 | 2.6E-84 | 2.5E-85 | 1.6E-91 | 1.2E-04 | 1.8E-120 | 3.8E-94 | 2.4E-138 | 7.5E-02 | 7.8E-120 | 0.0E+00 | 5.6E-47 | 8.2E-12 | NaN |

**Supplementary table 3.** p-values for Delong paired AUC comparisons, p16-HT-oof dataset

|  | No CST | CST1 | CST2 | CST3 | CST4 | CST5 | CST6 | CST7 | DA1 | DA2 | DA2_v2 | DA3 | DA4 |
| --- | --- | --- | --- | --- | --- | --- | --- | --- | --- | --- | --- | --- | --- |
| No CST | NaN | 3.29E-70 | 1.10E-02 | 1.39E-168 | 4.65E-66 | 1.24E-01 | 8.62E-01 | 2.00E-283 | 5.17E-04 | 9.09E-04 | 7.73E-04 | 1.66E-33 | 2.47E-14 |
| CST1 | 3.29E-70 | NaN | 7.53E-112 | 0.00E+00 | 1.17E-246 | 3.15E-104 | 8.00E-68 | 9.04E-147 | 3.86E-57 | 7.51E-80 | 4.07E-43 | 1.23E-152 | 1.68E-116 |
| CST2 | 1.10E-02 | 7.53E-112 | NaN | 1.41E-180 | 2.62E-66 | 3.08E-01 | 8.36E-03 | 0.00E+00 | 5.63E-10 | 2.93E-01 | 4.18E-09 | 2.84E-27 | 1.25E-08 |
| CST3 | 1.39E-168 | 0.00E+00 | 1.41E-180 | NaN | 3.35E-30 | 1.23E-154 | 7.52E-170 | 0.00E+00 | 2.56E-172 | 1.76E-141 | 4.02E-161 | 6.48E-51 | 3.56E-116 |
| CST4 | 4.65E-66 | 1.17E-246 | 2.62E-66 | 3.35E-30 | NaN | 5.91E-66 | 3.41E-79 | 0.00E+00 | 4.38E-89 | 3.62E-40 | 3.57E-87 | 5.05E-07 | 3.12E-30 |
| CST5 | 1.24E-01 | 3.15E-104 | 3.08E-01 | 1.23E-154 | 5.91E-66 | NaN | 7.45E-02 | 3.71E-306 | 2.59E-07 | 7.23E-02 | 2.15E-07 | 8.22E-28 | 4.04E-10 |
| CST6 | 8.62E-01 | 8.00E-68 | 8.36E-03 | 7.52E-170 | 3.41E-79 | 7.45E-02 | NaN | 0.00E-01 | 2.07E-03 | 1.85E-03 | 1.72E-03 | 1.30E-38 | 4.38E-15 |
| CST7 | 2.00E-283 | 9.04E-147 | 0.00E+00 | 0.00E+00 | 0.00E+00 | 3.71E-306 | 0.00E-01 | NaN | 2.01E-251 | 1.17E-284 | 7.02E-215 | 0.00E+00 | 0.00E+00 |
| DA1 | 5.17E-04 | 3.86E-57 | 5.63E-10 | 2.56E-172 | 4.38E-89 | 2.59E-07 | 2.07E-03 | 2.01E-251 | NaN | 8.16E-10 | 9.17E-01 | 1.58E-48 | 2.64E-24 |
| DA2 | 9.09E-04 | 7.51E-80 | 2.93E-01 | 1.76E-141 | 3.62E-40 | 7.23E-02 | 1.85E-03 | 1.17E-284 | 8.16E-10 | NaN | 4.30E-10 | 1.82E-19 | 5.78E-06 |
| DA2_v2 | 7.73E-04 | 4.07E-43 | 4.18E-09 | 4.02E-161 | 3.57E-87 | 2.15E-07 | 1.72E-03 | 7.02E-215 | 9.17E-01 | 4.30E-10 | NaN | 1.51E-48 | 1.29E-24 |
| DA3 | 1.66E-33 | 1.23E-152 | 2.84E-27 | 6.48E-51 | 5.05E-07 | 8.22E-28 | 1.30E-38 | 0.00E+00 | 1.58E-48 | 1.82E-19 | 1.51E-48 | NaN | 2.93E-10 |
| DA4 | 2.47E-14 | 1.68E-116 | 1.25E-08 | 3.56E-116 | 3.12E-30 | 4.04E-10 | 4.38E-15 | 0.00E+00 | 2.64E-24 | 5.78E-06 | 1.29E-24 | 2.93E-10 | NaN |

**Supplementary table 4.** p-values for Delong paired AUC comparisons, p16-XR dataset

|  | No CST | CST1 | CST2 | CST3 | CST4 | CST5 | CST6 | CST7 | DA1 | DA2 | DA2_v2 | DA3 | DA4 |
| --- | --- | --- | --- | --- | --- | --- | --- | --- | --- | --- | --- | --- | --- |
| No CST | NaN | 1.50E-23 | 0.00E+00 | 0.00E+00 | 1.19E-296 | 5.26E-175 | 0.00E+00 | 6.08E-01 | 2.19E-226 | 9.57E-129 | 1.50E-146 | 8.67E-39 | 3.63E-117 |
| CST1 | 1.50E-23 | NaN | 3.18E-182 | 4.70E-300 | 3.49E-191 | 3.51E-66 | 1.41E-274 | 3.09E-18 | 1.94E-106 | 9.94E-160 | 2.79E-46 | 1.15E-04 | 8.12E-43 |
| CST2 | 0.00E+00 | 3.18E-182 | NaN | 2.55E-114 | 2.57E-04 | 2.91E-71 | 2.32E-61 | 1.60E-234 | 1.42E-26 | 0.00E+00 | 8.06E-58 | 4.45E-119 | 1.29E-48 |
| CST3 | 0.00E+00 | 4.70E-300 | 2.55E-114 | NaN | 2.95E-98 | 3.32E-238 | 5.13E-14 | 0.00E+00 | 2.45E-177 | 0.00E+00 | 6.97E-218 | 0.00E+00 | 2.16E-246 |
| CST4 | 1.19E-296 | 3.49E-191 | 2.57E-04 | 2.95E-98 | NaN | 1.29E-77 | 3.14E-38 | 9.48E-292 | 5.79E-37 | 0.00E+00 | 2.94E-66 | 6.26E-165 | 2.60E-89 |
| CST5 | 5.26E-175 | 3.51E-66 | 2.91E-71 | 3.32E-238 | 1.29E-77 | NaN | 4.42E-182 | 2.11E-118 | 7.91E-09 | 0.00E+00 | 1.93E-01 | 1.46E-31 | 2.96E-01 |
| CST6 | 0.00E+00 | 1.41E-274 | 2.32E-61 | 5.13E-14 | 3.14E-38 | 4.42E-182 | NaN | 0.00E+00 | 1.39E-115 | 0.00E+00 | 2.62E-149 | 1.94E-232 | 1.15E-155 |
| CST7 | 6.08E-01 | 3.09E-18 | 1.60E-234 | 0.00E+00 | 9.48E-292 | 2.11E-118 | 0.00E+00 | NaN | 2.00E-153 | 2.97E-69 | 2.25E-87 | 6.66E-29 | 3.04E-106 |
| DA1 | 2.19E-226 | 1.94E-106 | 1.42E-26 | 2.45E-177 | 5.79E-37 | 7.91E-09 | 1.39E-115 | 2.00E-153 | NaN | 0.00E+00 | 9.77E-11 | 3.79E-55 | 1.50E-08 |
| DA2 | 9.57E-129 | 9.94E-160 | 0.00E+00 | 0.00E+00 | 0.00E+00 | 0.00E+00 | 0.00E+00 | 2.97E-69 | 0.00E+00 | NaN | 0.00E+00 | 9.01E-272 | 0.00E+00 |
| DA2_v2 | 1.50E-146 | 2.79E-46 | 8.06E-58 | 6.97E-218 | 2.94E-66 | 1.93E-01 | 2.62E-149 | 2.25E-87 | 9.77E-11 | 0.00E+00 | NaN | 9.05E-29 | 8.31E-01 |
| DA3 | 8.67E-39 | 1.15E-04 | 4.45E-119 | 0.00E+00 | 6.26E-165 | 1.46E-31 | 1.94E-232 | 6.66E-29 | 3.79E-55 | 9.01E-272 | 9.05E-29 | NaN | 7.29E-35 |
| DA4 | 3.63E-117 | 8.12E-43 | 1.29E-48 | 2.16E-246 | 2.60E-89 | 2.96E-01 | 1.15E-155 | 3.04E-106 | 1.50E-08 | 0.00E+00 | 8.31E-01 | 7.29E-35 | NaN |

**Supplementary table 5.** p-values for Delong paired AUC comparisons, p16-S360 dataset

|  | No CST | CST1 | CST2 | CST3 | CST4 | CST5 | CST6 | CST7 | DA1 | DA2 | DA2_v2 | DA3 | DA4 |
| --- | --- | --- | --- | --- | --- | --- | --- | --- | --- | --- | --- | --- | --- |
| No CST | NaN | 3.55E-175 | 0.00E+00 | 0.00E+00 | 0.00E+00 | 0.00E+00 | 0.00E+00 | 1.81E-38 | 0.00E-01 | 2.61E-20 | 2.93E-213 | 3.84E-109 | 9.25E-242 |
| CST1 | 3.55E-175 | NaN | 0.00E+00 | 1.21E-207 | 6.34E-228 | 5.64E-217 | 0.00E+00 | 1.80E-18 | 1.10E-43 | 7.50E-19 | 1.97E-10 | 1.67E-01 | 7.29E-31 |
| CST2 | 0.00E+00 | 0.00E+00 | NaN | 1.26E-21 | 5.83E-44 | 1.01E-66 | 1.16E-01 | 0.00E+00 | 2.85E-251 | 0.00E-01 | 0.00E+00 | 1.76E-257 | 7.82E-183 |
| CST3 | 0.00E+00 | 1.21E-207 | 1.26E-21 | NaN | 3.20E-04 | 5.56E-05 | 3.47E-18 | 2.87E-292 | 3.38E-98 | 3.50E-239 | 6.41E-198 | 5.75E-228 | 1.08E-124 |
| CST4 | 0.00E+00 | 6.34E-228 | 5.83E-44 | 3.20E-04 | NaN | 1.68E-01 | 2.17E-36 | 3.38E-260 | 1.42E-93 | 1.18E-194 | 4.30E-174 | 1.09E-215 | 8.58E-120 |
| CST5 | 0.00E+00 | 5.64E-217 | 1.01E-66 | 5.56E-05 | 1.68E-01 | NaN | 4.20E-41 | 3.17E-214 | 7.57E-88 | 2.95E-200 | 1.20E-149 | 7.37E-128 | 4.36E-59 |
| CST6 | 0.00E+00 | 0.00E+00 | 1.16E-01 | 3.47E-18 | 2.17E-36 | 4.20E-41 | NaN | 0.00E+00 | 4.07E-202 | 2.59E-277 | 3.20E-300 | 2.63E-252 | 2.01E-174 |
| CST7 | 1.81E-38 | 1.80E-18 | 0.00E+00 | 2.87E-292 | 3.38E-260 | 3.17E-214 | 0.00E+00 | NaN | 1.27E-74 | 1.94E-01 | 4.94E-39 | 4.83E-17 | 1.33E-72 |
| DA1 | 0.00E-01 | 1.10E-43 | 2.85E-251 | 3.38E-98 | 1.42E-93 | 7.57E-88 | 4.07E-202 | 1.27E-74 | NaN | 4.34E-62 | 2.13E-11 | 3.30E-18 | 5.69E-01 |
| DA2 | 2.61E-20 | 7.50E-19 | 0.00E-01 | 3.50E-239 | 1.18E-194 | 2.95E-200 | 2.59E-277 | 1.94E-01 | 4.34E-62 | NaN | 5.52E-44 | 7.96E-30 | 4.41E-76 |
| DA2_v2 | 2.93E-213 | 1.97E-10 | 0.00E+00 | 6.41E-198 | 4.30E-174 | 1.20E-149 | 3.20E-300 | 4.94E-39 | 2.13E-11 | 5.52E-44 | NaN | 2.56E-05 | 2.47E-12 |
| DA3 | 3.84E-109 | 1.67E-01 | 1.76E-257 | 5.75E-228 | 1.09E-215 | 7.37E-128 | 2.63E-252 | 4.83E-17 | 3.30E-18 | 7.96E-30 | 2.56E-05 | NaN | 1.63E-35 |
| DA4 | 9.25E-242 | 7.29E-31 | 7.82E-183 | 1.08E-124 | 8.58E-120 | 4.36E-59 | 2.01E-174 | 1.33E-72 | 5.69E-01 | 4.41E-76 | 2.47E-12 | 1.63E-35 | NaN |

**Supplementary table 6.** p-values for Delong paired AUC comparisons, CD3-HT dataset

|  | No CST | CST1 | CST2 | CST3 | CST4 | CST5 | CST6 | CST7 | DA1 | DA2 | DA2_v2 | DA3 | DA4 |
| --- | --- | --- | --- | --- | --- | --- | --- | --- | --- | --- | --- | --- | --- |
| No CST | NaN | 1.15E-04 | 9.72E-01 | 5.54E-94 | 9.90E-06 | 4.63E-01 | 7.68E-20 | 7.00E-119 | 1.87E-06 | 0 | 2.30E-17 | 1.55E-141 | 1.26E-87 |
| CST1 | 1.15E-04 | NaN | 7.39E-05 | 1.50E-83 | 2.01E-17 | 1.27E-03 | 3.48E-35 | 6.84E-93 | 8.50E-17 | 0 | 1.33E-06 | 5.52E-131 | 4.57E-75 |
| CST2 | 9.72E-01 | 7.39E-05 | NaN | 1.65E-134 | 4.90E-07 | 3.88E-01 | 8.24E-21 | 6.47E-119 | 2.29E-07 | 0 | 1.41E-23 | 4.00E-183 | 1.17E-118 |
| CST3 | 5.54E-94 | 1.50E-83 | 1.65E-134 | NaN | 3.60E-145 | 1.63E-128 | 1.18E-156 | 7.52E-01 | 1.23E-129 | 0 | 2.20E-76 | 3.62E-10 | 1.56E-04 |
| CST4 | 9.90E-06 | 2.01E-17 | 4.90E-07 | 3.60E-145 | NaN | 7.08E-09 | 1.31E-08 | 6.76E-151 | 8.94E-01 | 0 | 3.74E-44 | 4.25E-218 | 2.45E-148 |
| CST5 | 4.63E-01 | 1.27E-03 | 3.88E-01 | 1.63E-128 | 7.08E-09 | NaN | 5.43E-26 | 7.93E-117 | 7.87E-09 | 0 | 7.05E-20 | 7.13E-183 | 5.75E-114 |
| CST6 | 7.68E-20 | 3.48E-35 | 8.24E-21 | 1.18E-156 | 1.31E-08 | 5.43E-26 | NaN | 1.37E-225 | 9.69E-08 | 0 | 2.26E-59 | 4.97E-210 | 6.68E-151 |
| CST7 | 7.00E-119 | 6.84E-93 | 6.47E-119 | 7.52E-01 | 6.76E-151 | 7.93E-117 | 1.37E-225 | NaN | 1.33E-161 | 0 | 1.70E-61 | 6.08E-05 | 2.69E-03 |
| DA1 | 1.87E-06 | 8.50E-17 | 2.29E-07 | 1.23E-129 | 8.94E-01 | 7.87E-09 | 9.69E-08 | 1.33E-161 | NaN | 0 | 8.06E-42 | 2.32E-181 | 1.27E-129 |
| DA2 | 0.00E+00 | 0.00E+00 | 0.00E+00 | 0.00E+00 | 0.00E+00 | 0.00E+00 | 0.00E+00 | 0.00E+00 | 0.00E+00 | NaN | 0.00E+00 | 0.00E+00 | 0.00E+00 |
| DA2_v2 | 2.30E-17 | 1.33E-06 | 1.41E-23 | 2.20E-76 | 3.74E-44 | 7.05E-20 | 2.26E-59 | 1.70E-61 | 8.06E-42 | 0 | NaN | 6.02E-128 | 6.36E-61 |
| DA3 | 1.55E-141 | 5.52E-131 | 4.00E-183 | 3.62E-10 | 4.25E-218 | 7.13E-183 | 4.97E-210 | 6.08E-05 | 2.32E-181 | 0 | 6.02E-128 | NaN | 1.04E-25 |
| DA4 | 1.26E-87 | 4.57E-75 | 1.17E-118 | 1.56E-04 | 2.45E-148 | 5.75E-114 | 6.68E-151 | 2.69E-03 | 1.27E-129 | 0 | 6.36E-61 | 1.04E-25 | NaN |

**Supplementary table 7.** p-values for Delong paired AUC comparisons, CD8-HT dataset

|  | No CST | CST1 | CST2 | CST3 | CST4 | CST5 | CST6 | CST7 | DA1 | DA2 | DA2_v2 | DA3 | DA4 |
| --- | --- | --- | --- | --- | --- | --- | --- | --- | --- | --- | --- | --- | --- |
| No CST | NaN | 2.26E-02 | 1.12E-20 | 1.53E-02 | 1.93E-63 | 2.19E-01 | 6.40E-142 | 6.16E-105 | 5.63E-27 | 0 | 6.45E-04 | 6.95E-65 | 1.68E-18 |
| CST1 | 2.26E-02 | NaN | 2.28E-26 | 1.04E-04 | 3.85E-79 | 2.24E-01 | 4.71E-151 | 7.00E-94 | 1.79E-37 | 0 | 3.17E-01 | 1.20E-47 | 8.54E-11 |
| CST2 | 1.12E-20 | 2.28E-26 | NaN | 4.00E-07 | 6.01E-25 | 4.42E-34 | 1.06E-86 | 3.06E-171 | 4.00E-02 | 0 | 1.91E-37 | 1.28E-138 | 3.94E-66 |
| CST3 | 1.53E-02 | 1.04E-04 | 4.00E-07 | NaN | 1.09E-34 | 2.49E-04 | 8.69E-83 | 1.34E-107 | 1.47E-08 | 0 | 2.86E-08 | 2.08E-104 | 5.72E-34 |
| CST4 | 1.93E-63 | 3.85E-79 | 6.01E-25 | 1.09E-34 | NaN | 2.16E-92 | 1.55E-27 | 5.12E-226 | 1.07E-14 | 0 | 1.71E-93 | 1.45E-220 | 1.10E-142 |
| CST5 | 2.19E-01 | 2.24E-01 | 4.42E-34 | 2.49E-04 | 2.16E-92 | NaN | 1.90E-187 | 1.89E-108 | 2.90E-32 | 0 | 9.86E-03 | 4.57E-74 | 7.19E-19 |
| CST6 | 6.40E-142 | 4.71E-151 | 1.06E-86 | 8.69E-83 | 1.55E-27 | 1.90E-187 | NaN | 0.00E+00 | 4.46E-66 | 0 | 2.14E-159 | 1.24E-265 | 6.54E-184 |
| CST7 | 6.16E-105 | 7.00E-94 | 3.06E-171 | 1.34E-107 | 5.12E-226 | 1.89E-108 | 0.00E+00 | NaN | 1.10E-176 | 0 | 8.21E-81 | 6.86E-13 | 1.31E-45 |
| DA1 | 5.63E-27 | 1.79E-37 | 4.00E-02 | 1.47E-08 | 1.07E-14 | 2.90E-32 | 4.46E-66 | 1.10E-176 | NaN | 0 | 4.30E-38 | 1.30E-122 | 2.34E-59 |
| DA2 | 0.00E+00 | 0.00E+00 | 0.00E+00 | 0.00E+00 | 0.00E+00 | 0.00E+00 | 0.00E+00 | 0.00E+00 | 0.00E+00 | NaN | 0.00E+00 | 0.00E+00 | 0.00E+00 |
| DA2_v2 | 6.45E-04 | 3.17E-01 | 1.91E-37 | 2.86E-08 | 1.71E-93 | 9.86E-03 | 2.14E-159 | 8.21E-81 | 4.30E-38 | 0 | NaN | 1.99E-60 | 2.79E-11 |
| DA3 | 6.95E-65 | 1.20E-47 | 1.28E-138 | 2.08E-104 | 1.45E-220 | 4.57E-74 | 1.24E-265 | 6.86E-13 | 1.30E-122 | 0 | 1.99E-60 | NaN | 5.16E-28 |
| DA4 | 1.68E-18 | 8.54E-11 | 3.94E-66 | 5.72E-34 | 1.10E-142 | 7.19E-19 | 6.54E-184 | 1.31E-45 | 2.34E-59 | 0 | 2.79E-11 | 5.16E-28 | NaN |

**Supplementary table 8.** p-values for Delong paired AUC comparisons, all test sets

|  | No CST | CST1 | CST2 | CST3 | CST4 | CST5 | CST6 | CST7 | DA1 | DA2 | DA2_v2 | DA3 | DA4 |
| --- | --- | --- | --- | --- | --- | --- | --- | --- | --- | --- | --- | --- | --- |
| No CST | NaN | 3.69E-25 | 0.00E+00 | 1.44E-120 | 0.00E+00 | 3.29E-221 | 0.00E+00 | 1.62E-217 | 5.55E-284 | 0 | 5.54E-52 | 1.97E-205 | 1.01E-11 |
| CST1 | 3.69E-25 | NaN | 0.00E+00 | 7.06E-239 | 0.00E+00 | 0.00E+00 | 0.00E+00 | 5.60E-123 | 0.00E+00 | 0 | 1.35E-140 | 2.80E-119 | 1.49E-03 |
| CST2 | 0.00E+00 | 0.00E+00 | NaN | 9.02E-72 | 4.95E-102 | 3.13E-61 | 0.00E+00 | 0.00E+00 | 1.84E-29 | 0 | 8.28E-207 | 0.00E+00 | 0.00E+00 |
| CST3 | 1.44E-120 | 7.06E-239 | 9.02E-72 | NaN | 0.00E-01 | 6.61E-06 | 0.00E+00 | 0.00E+00 | 4.54E-13 | 0 | 4.68E-31 | 0.00E+00 | 0.00E-01 |
| CST4 | 0.00E+00 | 0.00E+00 | 4.95E-102 | 0.00E-01 | NaN | 1.66E-288 | 3.40E-99 | 0.00E+00 | 4.28E-240 | 0 | 0.00E+00 | 0.00E+00 | 0.00E+00 |
| CST5 | 3.29E-221 | 0.00E+00 | 3.13E-61 | 6.61E-06 | 1.66E-288 | NaN | 0.00E+00 | 0.00E+00 | 2.80E-04 | 0 | 2.81E-70 | 0.00E+00 | 0.00E+00 |
| CST6 | 0.00E+00 | 0.00E+00 | 0.00E+00 | 0.00E+00 | 3.40E-99 | 0.00E+00 | NaN | 0.00E+00 | 0.00E+00 | 0 | 0.00E+00 | 0.00E+00 | 0.00E+00 |
| CST7 | 1.62E-217 | 5.60E-123 | 0.00E+00 | 0.00E+00 | 0.00E+00 | 0.00E+00 | 0.00E+00 | NaN | 0.00E+00 | 0 | 0.00E+00 | 4.28E-04 | 3.98E-133 |
| DA1 | 5.55E-284 | 0.00E+00 | 1.84E-29 | 4.54E-13 | 4.28E-240 | 2.80E-04 | 0.00E+00 | 0.00E+00 | NaN | 0 | 2.81E-94 | 0.00E+00 | 0.00E+00 |
| DA2 | 0.00E+00 | 0.00E+00 | 0.00E+00 | 0.00E+00 | 0.00E+00 | 0.00E+00 | 0.00E+00 | 0.00E+00 | 0.00E+00 | NaN | 0.00E+00 | 0.00E+00 | 0.00E+00 |
| DA2_v2 | 5.54E-52 | 1.35E-140 | 8.28E-207 | 4.68E-31 | 0.00E+00 | 2.81E-70 | 0.00E+00 | 0.00E+00 | 2.81E-94 | 0 | NaN | 0.00E+00 | 3.50E-121 |
| DA3 | 1.97E-205 | 2.80E-119 | 0.00E+00 | 0.00E+00 | 0.00E+00 | 0.00E+00 | 0.00E+00 | 4.28E-04 | 0.00E+00 | 0 | 0.00E+00 | NaN | 2.18E-242 |
| DA4 | 1.01E-11 | 1.49E-03 | 0.00E+00 | 0.00E-01 | 0.00E+00 | 0.00E+00 | 0.00E+00 | 3.98E-133 | 0.00E+00 | 0 | 3.50E-121 | 2.18E-242 | NaN |
